# Charge-dependent modulation of S–H *vs.* O–H excited-state intramolecular proton transfer

**DOI:** 10.1039/d5sc10166b

**Published:** 2026-04-07

**Authors:** Chi-Chi Wu, Hao-Cheng Tsai, Hau-Yu Liu, Ya-Chen Lin, Chih-Hsing Wang, Alexander P. Demchenko, Chao-Tsen Chen, Pi-Tai Chou

**Affiliations:** a Department of Chemistry, National Taiwan University Taipei 10617 Taiwan R.O.C chenct@ntu.edu.tw chop@ntu.edu.tw; b Institute of Physical, Technical and Computer Sciences, Yuriy Fedkovych National University Chernivtsi 58002 Ukraine

## Abstract

Substituent effects critically influence electronic coupling and proton-transfer dynamics in excited-state intramolecular proton transfer (ESIPT), yet a quantitative link between charge redistribution and ESIPT behavior remains elusive. Here, we employ Natural Population Analysis (NPA) and the charge of the Substituent Active Region (cSAR) to quantify electronic responses for hydroxyl- *versus* thiol-functionalized flavonoids in ESIPT, enabling direct comparison with experimentally derived spectroscopic observations. Two distinct charge-redistribution regimes emerge: opposed shifts, in which donor and acceptor sites respond inversely to substitution, and concerted shifts, in which both sites gain charge in parallel. Thiol derivatives exhibit stronger substituent sensitivity and enhanced electronic polarization, whereas hydroxyl analogues display more limited yet directionally consistent charge responses across both donor and acceptor sites. Experimentally, this contrast results in a broader tunability of ESIPT rates in thiol systems, while hydroxyl analogues with similar substitution patterns show a narrower kinetic variation. Importantly, the excited-state donor and acceptor charges correlate far more strongly with cSAR(R″) than with classical Hammett *σ*_p_ parameters, underscoring the superior predictive power of cSAR in systems where conventional descriptors fail. Time-resolved fluorescence experiments corroborate the theoretical predictions, revealing a direct correlation between charge redistribution and the ESIPT rate. These findings establish the charge-based descriptors as predictive, mechanistic tools for understanding and designing ESIPT-active chromophores.

## Abstract 2

Substituent effects govern ESIPT through distinct charge-redistribution regimes. cSAR analysis reveals opposed and concerted charge shifts that control donor–acceptor polarization and predict ESIPT kinetics beyond classical descriptors.

## Graphical abstract

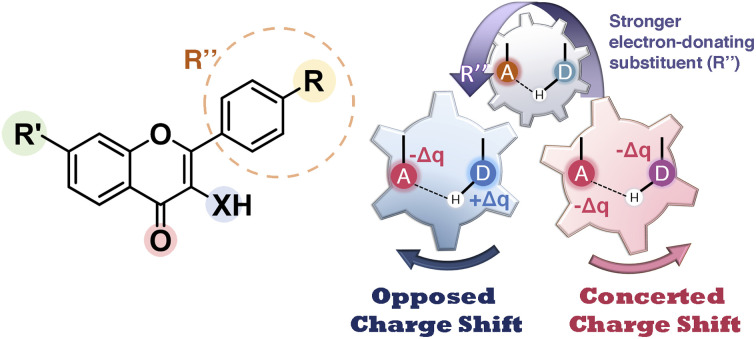

## Introduction

Excited-state intramolecular proton transfer (ESIPT) is a highly efficient photophysical process that produces a tautomeric species accessible only in the excited state, as its ground-state counterpart is too endergonic to be thermally populated relative to the normal form. Consequently, the resulting tautomer emission exhibits an anomalously large Stokes shift, defined by the onset separation from the normal absorption. In addition, molecular designs that promote coupling between ESIPT and excited-state intramolecular charge transfer (ESICT) have emerged as a powerful strategy to operate with this process.^1^ The ESIPT reactions have already achieved a broad range of applications, from OLEDs^2,3^ and lasers^4^ to scintillators^5^ and molecular sensors,^6^ and further progress is expected in new designs of the systems for transformation and conservation of energy.^7,8^ To deepen the understanding of proton-transfer reactions and stimulate further progress in this field, it is essential to explore new molecular systems and develop more refined analytical approaches. In this regard, sulfur atoms acting as ESIPT donors or acceptors have attracted particular interest. Due to their larger atomic size and higher electronic polarizability compared to conventional oxygen and nitrogen centers, sulfur-based hydrogen bonds and ESIPT processes exhibit distinctly different characteristics, as demonstrated in our previous studies.^9,10^ Our studies also demonstrate that the conventional theoretical frameworks used to describe ESIPT energetics and kinetics are frequently insufficient, especially in rationalizing how substituent effects alter the local charge distribution at the reaction centers. This challenge arises from a subtle yet critical interplay among donor acidity, acceptor basicity, hydrogen-bond strength, and solvent influence—factors that collectively govern the proton-transfer dynamics and the resulting photophysical behavior.^11–18^

**Fig. 1:**
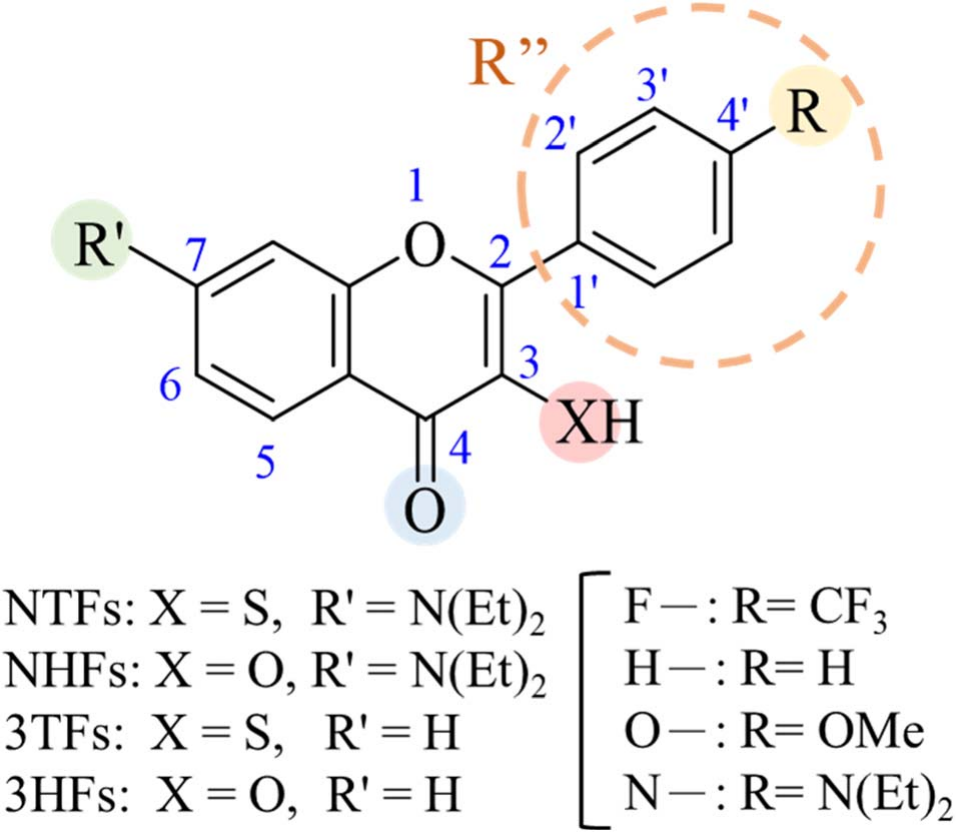
Molecular structures of flavonoid derivatives: 7-*N*,*N*-diethylamino-substituted thiolflavones (NTFs), 7-*N*,*N*-diethylamino-substituted hydroxyflavones (NHFs), C(4′)-substituted 3-thiolflavones (3TFs), and C(4′)-substituted 3-hydroxyflavones (3HFs). Substituents are defined as follows: R′ denotes the C(7) position; R represents the substituent on the C(4′) position (F– = CF_3_, H– = H, O– = OMe, and N– = N(Et)_2_); and for cSAR analysis, R″ corresponds to the entire C(4′) *para*-substituted phenyl ring (highlighted in the broken circle). The proton-donating group at C(3) (XH = SH or OH) defines each derivative series.

Traditional approaches for analyzing substituent effects, such as Hammett analysis, have yielded valuable insights into electronic perturbations in aromatic systems. However, Hammett *σ* constants are specifically defined for substituents on benzene rings and primarily capture inductive and resonance effects within that limited context.^19–23^ While effective for ground-state reactivity, these methods are less applicable to extended π-systems, cross-conjugated frameworks, or excited-state geometries. In ESIPT systems, substituent effects propagate through complex electronic pathways and often influence nonequilibrium geometries, making classical Hammett correlations insufficient to capture the full complexity of charge redistribution. These challenges are particularly evident in recently investigated sulfur-containing S–H flavone ESIPT systems, in which proton transfer takes place through the non-Pauling-type S–H⋯O=C hydrogen bond (H-bond) for a series of thiol flavones, including 7-*N*,*N*-diethylamino 3-mercaptoflavones (NTFs) and C(4′) substituted 3-thiolflavones (3TFs) (Fig. 1).^24–26^ In these studies, the corresponding substituent effects on ESIPT were evaluated using conventional Hammett correlations, which are commonly applied in O–H and N–H ESIPT systems. Such analyses typically relate O–H acidity and structural metrics to the rate trends of ESIPT.^16^ However, in S–H ESIPT systems, these approaches fail to rationalize the observed deviations from classical substituent trends. For example, in NTFs, C(4′)-substituents modulate tautomer emission primarily by altering the carbonyl acceptor basicity rather than by influencing the S–H bond strength.^10^ To further address this issue, we attempted to extend the investigation to other thiol flavone derivatives, 3TFs (Fig. 1). Unfortunately, most derivatives exhibit dominant nonradiative decay pathways arising from nπ*-dominated excited states, and only electron-rich substituents (*e.g.*, N(Et)_2_; see Fig. 1) retain sufficient ππ* character to yield measurable emission. On the other hand, the alternative method of transient absorption spectroscopy gives weak and complicated multiply overlapped spectra.^27^ These observations highlight the limitations of current experimental approaches.

To address these gaps, we first examined the full set of conventional ESIPT descriptors typically used to benchmark expected energetic and structural features, including excited-state potential energy surfaces (PESs) along the proton-transfer coordinate, excited-state reaction energies (Δ*E*^ES^_R_), and bond-length and vibrational analyses at the S_1_ minima. While comprehensive, these descriptors alone do not fully explain the substituent-dependent trends. We therefore introduce an independent, charge-based electronic perspective using two descriptors: (i) Natural Population Analysis (NPA) charges at the donor and acceptor sites and (ii) the charge of the Substituent Active Region (cSAR), which quantifies substituent-induced electron redistribution over the π-system.^28,29^ In contrast to geometric metrics, NPA directly probes how photoexcitation redistributes electronic density at the ESIPT-active moiety, while cSAR captures how a substituent electronically communicates with the proton-transfer core through the molecular framework. For example, substituents that induce similar hydrogen-bond geometries can nonetheless produce markedly different donor–acceptor charge responses, a distinction that is readily resolved by NPA and cSAR but obscured in conventional structural analyses. These electronic descriptors capture substituent effects not reflected in geometry alone and provide an alternative rationale for deviations from classical substituent trends.^20,28–31^ The detailed results and discussion are elaborated in the following sections.

## Results and discussion

### Concept of molecular design: selection of model ESIPT chromophores

To elucidate the distinct electronic roles of hydroxyl and thiol proton donors and to clarify how substituent effects propagate through the conjugated scaffold, a systematic set of ESIPT-active chromophores was examined. The study focuses on four flavonoid derivatives: 7-*N*,*N*-diethylamino-substituted thiolflavones (NTFs), 7-*N*,*N*-diethylamino-substituted hydroxyflavones (NHFs), C(4′)-substituted 3-thiolflavones (3TFs), and the corresponding unsubstituted 3-hydroxyflavones (3HFs) (see Fig. 1 for the structures). Each derivative is defined by variation at two key positions: substitution at the C(4′) position of the phenyl ring (R = CF_3_, H, OMe, or N(Et)_2_) and by the identity of the proton-donating group at C(3) (XH = SH or OH). This structural framework enables a controlled comparison of how donor identity, substituent-dependent electronic effects, and the chromanone core architecture collectively influence the charge redistribution and ESIPT behavior.

The choice of the above compounds is expected to resolve basic mechanisms of ESIPT functioning. The electronic characteristics of the donor atom are known to play a critical role in ESIPT. Oxygen, with compact 2p orbitals and high electronegativity, exerts a strong inductive electron-withdrawing effect and engages efficiently in 2p–2p π-overlap with adjacent aromatic centers. Its lone pairs readily delocalize into the π-system, providing resonance stabilization and contributing electron density to the aromatic scaffold. As a result, O–H-containing systems are commonly described as exhibiting a positively polarized ipso carbon and a partially negatively charged oxygen atom, features that have been widely discussed in the context of O–H ESIPT systems.^1,32^

Sulfur, in contrast, exhibits lower electronegativity and larger, more diffuse 3p orbitals. The resulting 3p–2p orbital mismatch reduces π-conjugation efficiency with the aromatic framework and weakens classical inductive effects, despite sulfur's higher polarizability.^33,34^ These electronic distinctions have been invoked to rationalize the unconventional hydrogen-bonding and proton-transfer behavior observed in sulfur-containing ESIPT chromophores.^10,27^ Within this context, our charge analysis indicates that thiol-containing systems exhibit a more negatively polarized ipso carbon and a partially positive sulfur center, an electronic arrangement that enhances the effective acidity of the S–H proton. Consequently, it could be suggested that, in S–H ESIPT systems, proton-transfer kinetics are governed less by intrinsic S–H bond strength and more by the basicity of the acceptor site. In the present flavone scaffold, this acceptor strength is modulated primarily by the 7-*N*,*N*-diethylamino substituent, which tunes the electronic environment of the carbonyl group and thereby controls ESIPT dynamics.

In contrast, the hydroxyl-based systems possess lower donor acidity, and their ESIPT efficiency depends on the cooperative interplay between O–H bond strength and carbonyl basicity.^35^ Based on this distinction, thiol-containing chromophores are expected to be particularly sensitive to modulation of the carbonyl acceptor through C(7) substitution, whereas hydroxyl-containing analogues likely require simultaneous tuning of both donor bond strength and acceptor character to achieve efficient proton transfer.

We note that nuclear (hydrogen atom) tunnelling and vibronic coupling may, in principle, contribute to the ESIPT process. In the hydroxyflavones (3HFs and NHFs), the strong coupling to the π-system is expected to limit tunnelling contributions to the substituent-dependent trends. In the thiolflavones (3TFs and NTFs), the heavier sulfur donor may further suppress tunnelling. Accordingly, the use of classical S_1_ potential-energy surfaces and charge-based descriptors provides an appropriate framework for analyzing substituent-controlled ESIPT behavior.

### Experimental ESIPT spectroscopy and proton-transfer kinetics

Experimentally, the trend of ESIPT dynamics of NTFs, NHFs, 3TFs, and 3HFs differs markedly. Note that the NTFs and 3TFs were reported previously,^9,10^ whereas the NHFs and 3HFs were synthesized and characterized in this work (Fig. S1–S13). Steady-state fluorescence spectra of 3HFs and NHFs are shown in Fig. S14, while the corresponding time-resolved kinetic spectra are presented in Fig. S15. Details of the syntheses and characterization are provided in the SI. The NTF series comprises four *para*-substituted derivatives (F-, H-, O-, and N-NTF) designed to modulate the electron density across the π-system. The time-resolved fluorescence measurements reveal fast proton transfer, with *k*_PT_ values ranging from 8.2 ps^−1^ to 2.3 ps^−1^ and an experimentally observed rate order of N- > O- > H- > F-NTF.^10^ The 3TFs, also synthesized in the previous study,^27^ exhibit a different behavior. Although computations predict nearly barrierless ESIPT, experimental confirmation is limited because the tautomeric S_1_ state of most 3TFs is dominated by nonradiative decay. Both H-3TF and F-3TF display no measurable emission due to their predominant nπ* character, which promotes rapid internal conversion. Only N-3TF, in which the N(Et)_2_ substituent enhances π-delocalization and preserves ππ* character in the tautomer, exhibits a distinct emission band at 710 nm.

In contrast, the NHFs and 3HFs synthesized here exhibit slower ESIPT overall, with rate constants summarized in Table S6. For NHFs, the rate trend follows O- > N- > H-NHF ∼ F-NHF. For 3HFs, the corresponding trend is N- > F- > H-3HF ≈ O-3HF. Two systems, F-NHF and N-3HF, show slight deviations from these trends. Their distinct charge distributions render the normal (N*) form sensitive to solvent polarity. As solvent polarity increases, N* is preferentially stabilized, lowering its energy, and increasing the proton-transfer barrier.^36,37^ Consequently, the ESIPT rate decreases, and a larger fraction of the steady-state fluorescence originates from the non-transferred N* population.

As clearly shown in Table 1, experimental ESIPT kinetics of the studied four classes of compounds reveal significant substituent effects. While these observables alone do not reveal the electronic origin of the trends, the following section on charge redistribution and substituent-controlled electronic response demonstrates that the observed kinetics are consistent with the computed electronic-structure descriptors (NPA charges and cSAR values), providing mechanistic insight into how substituents modulate the ESIPT process (*vide infra*). A detailed kinetic modelling approach based on first-principles potential-energy surface scans could provide a more quantitative comparison, but this is beyond the scope of the present study and could be pursued in future work.

**Table 1:** Proton-transfer time constants (*τ* (ps)) of NHFs, 3HFs, and NTFs^10^ that were determined by fluorescence upconversion at the first absorption band in dilute toluene. F, H, O, and N indicate the substituents on the C(4′) phenyl ring

Series of compounds	F-*τ* (ps)	H-*τ* (ps)	O-*τ* (ps)	N-*τ* (ps)
NHFs	4.2	3.7	1.3	1.7
3HFs	0.8	0.75	0.69	2.8
NTFs	0.43	0.29	0.15	0.12

### Conventional ESIPT descriptors: energetics and structural parameters

To benchmark these trends against established frameworks, the conventional ESIPT descriptors, including potential energy surfaces (PES), reaction energies (Δ*E*^ES^_H_), bond lengths, and vibrational frequencies at S_1_-opt structures, were examined. The electronic configurations of all species are presented in Fig. S15 and S16 along with corresponding orbital analyses in Fig. S19, S20, and in Table S1. In all compounds, the tautomeric S_1_(T) state is the most stable excited-state minimum, confirming the favorable thermodynamics of ESIPT. 7-*N*,*N*-Diethylamino-substituted systems (NTFs and NHFs) display ππ* character in both S_1_(N) and S_1_(T), consistent with the delocalizing effect of the –N(Et)_2_ group at C(4′), which enhances conjugation across the scaffold.

The potential energy surfaces (PES) analysis in toluene, illustrated in Fig. S22, provide illustrative paths along the proton vibration vector, offering a qualitative view of ESIPT kinetics. H-3TF and H-NTF show effectively barrierless profiles (Δ*E*^‡^ ≈ 0.04–0.06 eV), whereas H-NHF and H-3HF exhibit higher barriers (0.11–0.21 eV). In all cases, S_1_(T) lies below S_1_(N), but PES barriers alone do not fully differentiate substituent effects, especially in weakly polarized systems. The excited-state reaction energy, Δ*E*^ES^_R_ = *E*_N*_ − *E*_T*_, serves as a thermodynamic indicator of ESIPT propensity, particularly when barriers are small.^12,38,39^ Trends within each series (Table S2) broadly follow experimental behavior—for example, in NTFs, Δ*E*^ES^_R_ becomes increasingly exothermic from F- < H- < N- < O-NTF, paralleling the measured rate constants. Similar correlations occur in NHFs, whereas the trends in 3TFs and 3HFs are noticeably ambiguous. In these scaffolds, the excited states often contain substantial nπ* character or involve competing nonradiative decay pathways, which obscure the expected correlation between substituent effects and charge redistribution.

Structural descriptors (C=O, X–H bond lengths and vibrational frequencies) listed in Tables S3 and S4 supplement the energetic picture. Substituents modulate X–H acidity and C=O basicity upon excitation, as reflected by Δ(S–H) and Δ(C=O) values between S_0_ and S_1_. In NTFs, Δ(C=O) increases in the order of F- < H- < O- < N-NTF, matching the trend in *k*_PT_. NHFs show analogous but weaker substituent dependence. In contrast, 3TFs and 3HFs display inconsistent correlations: Δ(X–H) values are small or irregular, and Δ(C=O) trends do not reflect the actual ESIPT outcomes. In 3HFs, particularly, ESIPT is not detected experimentally even though Δ(C=O) changes systematically with substitution. In brief, conventional ESIPT descriptors capture general substituent influences, but they do not provide a unified electronic rationale for the observed kinetic trends. This gap motivates the introduction of an alternative electronic-response framework presented in the next section, which constitutes the core of this study.

### Charge redistribution and substituent-controlled electronic response

The limitations of conventional descriptors appear because charge redistribution is the primary origin of substituent-dependent ESIPT behavior, and it was not properly accounted for. Since proton transfer responds directly to the electronic polarization along the donor–H–acceptor axis, the substituent-induced changes in local charge and field asymmetry provide a more physically transparent and mechanistically grounded explanation than that based on energetics only.

Natural Population Analysis (NPA) allows characterizing the substituent-induced electronic perturbations propagating through the molecular framework and the redistribution of charge between the proton donor and acceptor sites in the excited state. The resulting charge redistribution patterns reveal two characteristic electronic response modes among the studied ESIPT chromophores, NTFs, NHFs, 3TFs, and 3HFs, reflecting how substituent perturbations are transmitted through the molecular scaffold. The results shown in Fig. S23, reveal that NTFs and NHFs exhibit opposed charge shift behavior, in which electron-donating substituents at the C(7) and C(4′) positions induce antagonistic responses at the donor and acceptor sites, leading to enhanced donor–acceptor polarization along the ESIPT coordinate. In contrast, 3TFs and 3HFs display concerted charge shift patterns, in which donor and acceptor sites respond to substitution in the same direction. These distinct redistribution patterns reflect differences in the connectivity of the π-electronic framework that mediates substituent perturbations.

**Fig. 2:**
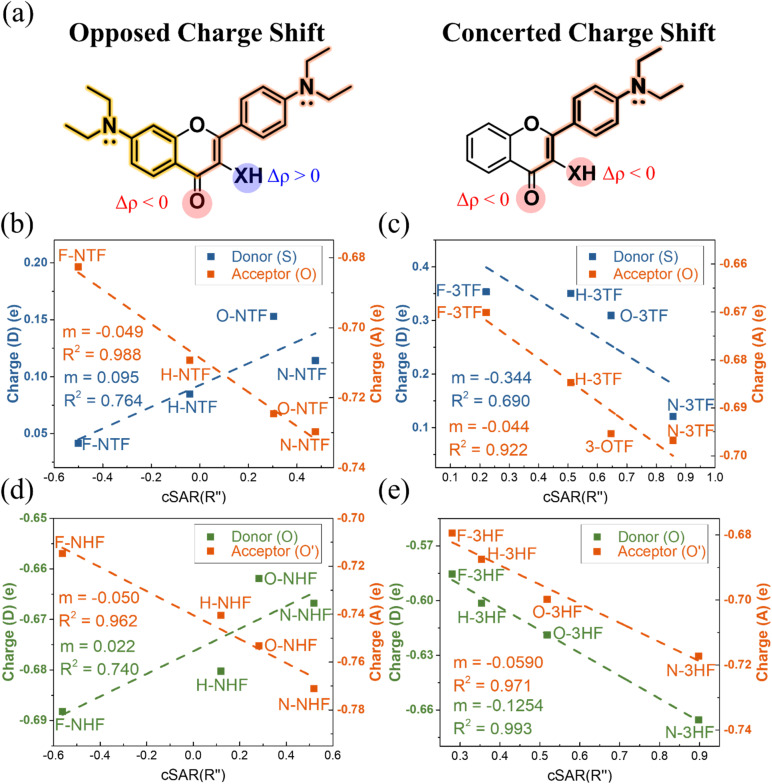
(a) Schematic illustration of substituent-induced charge-flow pathways and their impact on donor–acceptor charge redistribution in flavonoid chromophores. The backbone colors denote the origin of electronic communication: yellow indicates C(7)-mediated long-range charge flow enabled by extended π-delocalization, whereas orange represents direct C(4′)-substituent-induced charge flow. Charge redistribution at the proton-transfer moiety is encoded by color highlights, with red indicating electron accumulation at the acceptor (Δ*ρ*(A) < 0) and blue indicating electron depletion at the donor (Δ*ρ*(D) > 0). The proton donor is denoted as X = O or S. In NTFs/NHFs, the coexistence of C(7)- and C(4′)-mediated pathways produces an opposed charge-shift response, while in 3TFs/3HFs the absence of the C(7) pathway results in a concerted donor–acceptor response. (b–e) Plots of substituent-active region charge (cSAR) *versus* Natural Population Analysis (NPA) charges on ESIPT donor (left-axis) and acceptor (right-axis) atoms for (b) NTFs, (c) NHFs, (d) 3TFs, and (e) 3HFs. Donor atoms (S or O) are colored blue (S) or green (O); acceptor O/O′ atoms are orange. Dashed lines denote linear regressions, with slopes (*m*) and *R*^2^ values indicated.

Fig. 2(a) illustrates schematically that the scaffold connectivity allowing polarization of the π-electronic system provides a strong impact exceeding that arising from the identity of the donor atom alone.

In NHFs and NTFs, the C(7) *N*,*N*-diethyl group enables an additional C(7)-mediated long-range charge-flow pathway through resonance and inductive contributions, extending the π-system and enabling coupling between distant substituents and the donor–acceptor sites. The coexistence of this C(7)-mediated pathway with direct C(4′)-induced charge flow produces the opposed charge-shift regime characterized by enhanced donor–acceptor polarization along the ESIPT coordinate.

In contrast, 3TFs and 3HFs lack this extended delocalization pathway. As a result, substituent effects propagate more symmetrically through the molecular framework, leading to a concerted charge-shift regime, in which donor and acceptor sites respond in the same direction. This regime corresponds to a more balanced redistribution of electron density between the donor and acceptor sites and therefore a less strongly polarized donor–acceptor axis. The electronic origin of these distinct charge-flow pathways is further illustrated by the resonance structures shown in Fig. S24.

While H-3HF appears to be ESIPT kinetically favorable based on shallow PES barriers, as discussed in the previous section, mechanistic insight emerges when considering the charge redistribution patterns. H-NTFs and H-NHFs, which operate in opposed charge-shift systems and exhibit strong donor–acceptor polarization, stabilize the proton-transferred tautomer and help compensate for modest PES barriers. In contrast, H-3TF, despite its low PES barrier, belongs to the concerted regime with weaker donor–acceptor polarization, which may limit proton transfer efficiency. These observations underscore the importance of quantifying local electrostatic effects, as analyzed below through donor/acceptor site fields and potentials, and their correlations with experimental ESIPT rates.

The charge of the Substituent Active Region (cSAR(R″)) was then calculated to quantify substituent influence, treating the entire C(4′)-substituted phenyl ring as the functional group (R″). This approach accounts for both inductive and resonance contributions as they are distributed through the π-framework. Substituents ranged from electron-withdrawing (–CF_3_) to electron-donating groups (–OCH_3_ and –N(Et)_2_), and for comparative purposes, their classical *para*-Hammett constants (*σ*_p_) were also evaluated, with all substituent descriptors summarized in Fig. S25.

Plots of donor and acceptor site charges *versus* cSAR(R″) are shown in Fig. 2(b)–(e). In NTFs and NHFs, the donor and acceptor charges correlate strongly, but in opposite directions, with cSAR(R″), reflecting effective donor–acceptor polarization and directional charge redistribution. This trend is especially pronounced in NTFs, where sulfur's reduced electron-withdrawing ability amplifies its responsiveness to remote substituents. NHFs exhibit similar behavior, though the effect is partially moderated by strong ground-state hydrogen bonding. In contrast, 3TFs and 3HFs demonstrate correlations of the same sign at both donor and acceptor sites, consistent with more uniform charge accumulation.

Notably, 3TFs exhibit greater substituent sensitivity at the donor site than 3HFs, which is consistent with sulfur's weaker covalent bond strength and its more flexible polarizability. Importantly, the excited-state donor and acceptor charges exhibit stronger correlations with cSAR(R″) than with Hammett *σ*_p_ values shown in Fig. S26 and S27, underscoring the relevance of cSAR for analysing mechanisms in systems where conventional descriptors fail.

To elucidate the relationship between charge redistribution and ESIPT kinetics, the local electric field (*F*_LE_) and electrostatic potential (*V*_ES_) at the donor and acceptor sites were evaluated from NPA atomic charges and quantitatively correlated with the experimental rate constants. The results obtained with the expressions for *F*_LE_ and *V*_ES_ provided in eqn (3) and (4) in the computational section are shown in Fig. S28. The absolute values, *F*_LE_(D), *F*_LE_(A), *V*_ES_(D), and *V*_ES_(A), together with the differences Δ*F*_LE_ and Δ*V*_ES_, capture the extent of electrostatic polarization along the D–H⋯A axis.

Among these descriptors, Δ*F*_LE_ and Δ*V*_ES_ emerge as the most illustrative and kinetically relevant quantities. By definition, the local electric field is the negative gradient of the electrostatic potential, *F*_LE_ = −∇*V*_ES_, which directly reflects the slope of the potential experienced by the proton; larger asymmetry therefore denotes a stronger local driving force for the transfer. In the opposed charge-shift systems, NTFs, both Δ*F*_LE_ and Δ*V*_ES_ exhibit systematic monotonic trends with ln(*k*_PT_), indicating that the directional electronic polarization between the donor and acceptor accelerates ESIPT. In NHFs, the correlations are noticeably weaker and less systematic, consistent with a more delocalized and less directional charge redistribution that reduces the effectiveness of electrostatic driving along the reaction coordinate. In 3HFs demonstrating the concerted behavior, linearity is maintained for moderate substituents (–CF_3_, –H, and –OMe), while the strongly donating –N(Et)_2_ induces nonlinear polarization at high electron density. Intrinsic donor/acceptor characteristics further modulate the trends: thiol systems, dominated by carbonyl basicity, show tighter links between electrostatic descriptors and rate constants, whereas hydroxyl systems depend on a balance of donor acidity and acceptor basicity, yielding more moderate sensitivities.

Additional calculations using CAM-B3LYP and ωB97X-D (Tables S6–S9) confirm that the acceptor site becomes more negative with increasingly electron-donating substituents, consistent with the B3LYP results. In the NTFs, however, the donor sulfur charge shows greater sensitivity to the treatment of long-range exchange, leading to quantitative differences in the predicted trend. Despite these variations, the frontier orbital distributions and the S_1_ excited-state character remain qualitatively similar across functionals, indicating that the underlying electronic structure is preserved. Notably, the B3LYP calculations reproduce the experimentally observed absorption and emission wavelengths and their substituent-dependent trends, supporting the mechanistic interpretation derived from the B3LYP NPA analysis. The functional dependence therefore primarily affects the magnitude of the predicted polarization rather than the identification of the opposed and concerted charge-shift regimes.

**Fig. 3:**
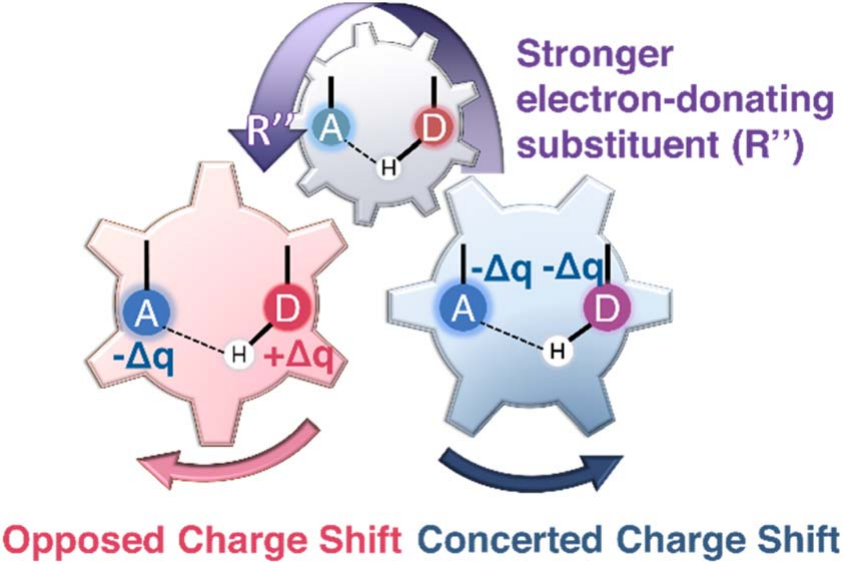
Schematic illustration of the two substituent-controlled electronic charge redistributions governing ESIPT in flavonoid chromophores. Increasing electron donation from the substituent R″ (indicated by the arrow) induces either an opposed charge-shift regime, in which Δ*q*_D_ and Δ*q*_A_ change in opposite directions, or a concerted shift, in which both sites respond in the same direction. These “gear-like” electronic responses account for the distinct substituent sensitivities and photophysical behaviors of the studied NTFs/NHFs and 3TFs/3HFs.

Together, these results show that substituent-induced electronic polarization, captured by cSAR(R″), NPA charges, and local electrostatic descriptors, provides a mechanistically consistent explanation for ESIPT reactivity within the experimentally accessible flavonoid chromophores. As summarized in Fig. 3, the electron-donor substituent R″ that involves a polarizable phenyl ring serves as an electronic “control gear”, producing either opposed (NTFs and NHFs) or concerted (3TFs and 3HFs) charge-shift regimes that dictate substituent sensitivities and photophysical behavior.

## Conclusion

In summary, this work provides a unified electronic framework for understanding substituent-dependent ESIPT across thiol- and hydroxyl-functionalized flavonoids. While the donor identity and scaffold connectivity establish the baseline acidity/basicity balance, experimental kinetics and photophysical data reveal that the substituent electronics ultimately govern the efficiency of proton transfer. It becomes evident that conventional descriptors (PES barriers, reaction energies, bond length changes, and vibrational shifts) capture global thermodynamic trends but do not fully account for the diverse kinetic behaviors observed across the series of ESIPT dyes. Instead, the charge-based descriptors offer a more physically and mechanistically transparent interpretation.

The NPA charges and cSAR(R″) quantify two distinct electronic response modes: an opposed charge-shift regime (NTFs and NHFs) and a concerted regime (3TFs and 3HFs). Analysis of local electrostatic fields and potentials directly links these charge-redistribution patterns to measured ESIPT rate constants, demonstrating that directional polarization along the D–H⋯A axis, rather than static structure or simple energetics, dictates proton-transfer efficiency. Comparisons between thiol (S–H) and hydroxyl (O–H) systems reveal that S–H donors, due to larger, more polarizable 3p orbitals, generate stronger donor–acceptor polarization and are primarily sensitive to modulation of the carbonyl acceptor. In contrast, O–H donors, with compact 2p orbitals and higher intrinsic acidity, require cooperative tuning of both donor bond strength and acceptor basicity to achieve efficient ESIPT.

Overall, charge redistribution emerges as a robust predictor of ESIPT reactivity in these systems. This electronic-response framework provides actionable design principles for tailoring ESIPT rates and emission properties, offering guidance for the development of functional excited-state proton-controlled materials.

### Computational details

All calculations were performed using the Gaussian 16 software package in version G16RevC.02.^40–42^ Geometry optimizations and electronic-structure analyses were primarily carried out using the B3LYP hybrid exchange–correlation functional with the 6-311++G(3df, 3pd) basis set. The B3LYP functional was selected as the primary method because it reproduces the experimentally observed absorption and emission wavelengths and their substituent-dependent trends for these systems. Unless otherwise specified, all reported S_1_ properties and discussions are based on results obtained at the B3LYP level of theory. To assess the sensitivity of the results to the choice of the functional, selected calculations were also performed using the long-range corrected CAM-B3LYP functional, and, for the 3TFs, the range-separated ωB97X-D functional was used.^43,44^ The resulting electronic structures and charge-transfer characteristics were found to be qualitatively consistent across these functionals. For NHF and NTF systems, ωB97X-D calculations were not fully converged due to computational resource limitations, but CAM-B3LYP results provide a consistent description of the electronic structure and mechanistic trends (Tables S6–S9).

All experiments were conducted in nonpolar or weakly polar solvents, namely cyclohexane and toluene. Accordingly, the calculations and related discussions are based on a toluene environment (*ε* = 2.38) using the Polarizable Continuum Model (PCM), to avoid the influence of highly polar media, which could induce substantial external reorganization energy and thereby introduce an additional reaction barrier.^45,46^ Natural Transition Orbital (NTO) analysis was carried out to characterize the electronic nature of the S_1_ → S_0_ transitions. By reducing complex excitations to the dominant hole–particle pairs, NTOs provide a compact and physically meaningful representation of excitation character and potential charge-transfer features.^47^

Charge distributions were evaluated using the Natural Population Analysis (NPA), which improves upon the basis-set sensitivity of Mulliken population analysis by providing more chemically meaningful atomic charges. NPA is based on the Natural Bond Orbital (NBO) framework developed by Foster and Weinhold,^48^ wherein a set of orthonormal natural atomic orbitals (NAOs) is constructed from the system's one-particle density matrix. The atomic charges are then obtained from the occupancies of these NAOs. This method yields numerically stable and physically interpretable charge distributions, particularly in systems with high ionic character or extensive conjugation.^28,49–51^ NPA charges and NAOs were obtained using the built-in NBO analysis as implemented in Gaussian. Natural transition orbitals (NTOs) were also generated from TD-DFT calculations using the Gaussian package. All electronic descriptors reported in this work were evaluated in the S_1_-optimized geometries, ensuring that the analysis reflects the excited-state electronic structure rather than ground-state properties. To quantify the electronic influence of substituents across the flavonoid framework, the Hammett model and the charge of the Substituent Active Region (cSAR) were utilized.^29^ Both methods quantify substituent-induced electronic perturbations, yet they differ fundamentally in origin and scope. In the Hammett model, reaction kinetics or equilibria for a substituted benzoic acid derivative are related to those of the parent compound *via*the following equation:^23^

1 log *k* = log *k*_0_ + *ρσ*

where *k* and *k*_0_ are reaction rate constants for a substituted and an unsubstituted benzoic acid derivative, respectively. *σ* represents an experimentally determined substituent constant, quantifying the electronic effect of the substituent. At the same time, *ρ* is a constant that depends on the reaction mechanism and environment and reflects the reaction's sensitivity to electronic effects. Although this relation elegantly partitions inductive and resonance contributions, its reliance on empirical correlations and neglect of steric and conformational nuances limit predictive fidelity, particularly in non-standard environments or excited-state processes.

By contrast, cSAR delivers a physically grounded measure of local electronic influence across any molecular framework. The cSAR value is defined as follows:^30^

2 cSAR(R) = *q*(Sub) + *q*(C_ipso_)

Here, *q*(S) is the charge of the substituent (Sub), and *q*(C_ipso_) is the atomic charge of C_ipso_, *i.e.*, the carbon atom directly substituted with Sub. Intuitively, a negative cSAR(Sub) value indicates that Sub has an electron-withdrawing effect, while a positive value suggests that it has an electron-donating effect. Because cSAR arises directly from electronic structure calculations, it inherently captures both geometric and electronic context, making it applicable to the ground and excited states; in the present work, all cSAR values are derived from excited-state (S_1_) electronic densities.

In this work, substituent effects are analyzed using a unified notation for the flavonoid scaffold shown in Fig. 1. The substituent at the C(7) position is denoted as R′, while R refers to the terminal *para*-group attached to the C(4′) position. For quantitative analysis using cSAR, the effective substituent is defined as R″, which corresponds to the entire C(4′) *para*-substituted phenyl ring rather than only its terminal functional group. This extended definition accounts for both resonance and inductive contributions transmitted through the π-conjugated system, enabling a more complete description of the substituent-induced electronic response across the ESIPT chromophore.

To extract the essential electrostatic factors that influence proton transfer, the local electric field (*F*_LE_) and electrostatic potential (*V*_ES_) descriptors were constructed using only the donor atom (D: S or O), acceptor atom (A: O), and transferring proton (H) atoms.^52^ This simplified three-center model captures intrinsic coulombic interactions within the critical hydrogen-bonding motif while intentionally excluding the long-range charge effects from the extended molecular structure. Atomic charges were determined from NPA at the S_1_-opt geometries. For each D and A, the electrostatic potential at the proton position was calculated as follows:3
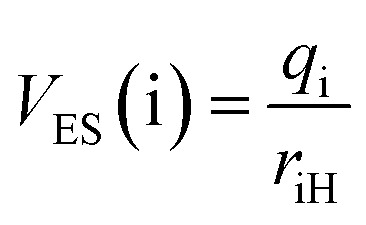
where *q*_i_ is the NPA charge on atom i (D or A), and *r*_iH_ is the distance between the proton H and atom i. The corresponding local electric field projected along the donor–acceptor axis was evaluated as follows:4
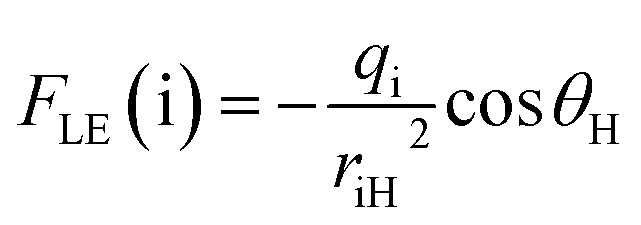
where *θ*_H_ represents the angle between the donor–acceptor axis and the vector connecting atom i to H.^12^ The site-specific terms, *F*_LE_(D), *F*_LE_(A), *V*_ES_(D), and *V*_ES_(A), represent the donor and acceptor influences at the proton position. Their differences, Δ*F*_LE_ = *F*_LE_(D) − *F*_LE_(A) and Δ*V*_ES_ = *V*_ES_(D) − *V*_ES_(A), quantify the local electrostatic asymmetry driving proton transfer. All calculations utilized in-house scripts for postprocessing NPA charge outputs according to these equations.

## Author contributions

C.-C. Wu performed the theoretical calculations and electronic structure analyses. H.-Y. Liu, Y.-C. Lin, and C.-H. Wang conducted the spectroscopic experiments. H.-C. Tsai was responsible for the synthesis of the compounds. A. P. Demchenko provided valuable comments and suggestions during manuscript preparation. C.-T. Chen and P.-T. Chou initiated and supervised the project. All authors contributed to the preparation of the manuscript.

## Conflicts of interest

There are no conflicts to declare.

## Supplementary Material

SC-OLF-D5SC10166B-s001

## Data Availability

Supplementary information (SI): details of synthetic procedures and additional NMR spectra, computational approach, and supporting photophysical measurements. See DOI: https://doi.org/10.1039/d5sc10166b.
