# Supplementary material for: Charge-dependent modulation of S–H vs. O–H excited-state intramolecular proton transfer
Source: Chem Sci. 2026 Apr 7;17(22):10982–91. doi: 10.1039/d5sc10166b (PMC13103892; doi:10.1039/d5sc10166b)
Supplement: SC-017-D5SC10166B-s001 [file SC-017-D5SC10166B-s001.pdf]

Supplementary Information (SI) for Chemical Science.
This journal is © The Royal Society of Chemistry 2026

Supporting Information

Charge-Dependent Modulation of S–H vs O–H Excited-State Intramolecular Proton Transfer

Chi-Chi Wu,[1#] Hao-Cheng Tsai,[1#] Hau-Yu Liu,[1] Ya-Chen Lin,[1] Chih-Hsing Wang,[1] Alexander P. Demchenko,[2] Chao-Tsen Chen,*[1] Pi-Tai Chou*[1]

[1]Department of Chemistry, National Taiwan University, Taipei, 10617, Taiwan, R.O.C.

[2]Institute of Physical, Technical and Computer sciences, Yuriy Fedkovych National University, Chernivtsi, 58002, Ukraine.

[#] equal contribution

Experimental Information

1. General Experimental Section

All chemicals were purchased from commercial sources and used as received. Solvents used for syntheses were used without drying. Merck silica gel 60 F254 was used as TLC plate, visualized by UV light. ^{1}H (400 MHz) and ^{13}C (100 MHz) spectra were recorded on a Bruker AVIII 400 spectrometer. Chemical shifts are reported relative to $CDCl_3$ (7.24 ppm for ^{1}H, 77.00 ppm for ^{13}C) and quoted as δ values in ppm. All NMR spectra are recorded at ambient temperature unless otherwise specified. The following abbreviations are used: singlet (s), doublet (d), triplet (t), quartet (q), multiplet (m), and broad (br). Melting points were determined by using the Fargo MP-1D melting point apparatus without correction. Infrared spectra were recorded on a Varian 640-IR spectrometer. Mass spectra with an electrospray ionization (ESI) were determined on a Bruker microTOFQⅡ spectrometer.

2. Experimental Procedures

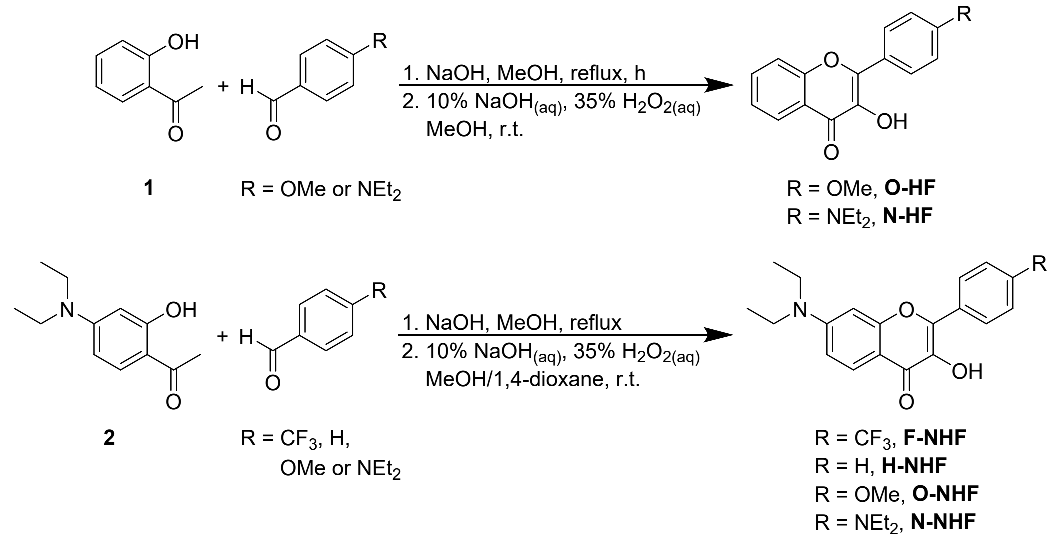

3HFs including **O-3HF**, **N-HF** and all **NHFs** were synthesized *via* Claisen-Schmidt condensation followed by Algar-Flynn-Oyamada reaction under modified conditions according to reported procedures. [1,2]

The starting material compound **1** and all corresponding aldehydes were purchased from commercially available source. Compound **2** (1-(4-diethylamino-2-hydroxy-phenyl)ethan-1-one) was synthesized according to previously reported procedures.[3]

3-hydroxy-2-(4-methoxyphenyl)-4H-chromen-4-one (O-3HF)

Pale yellow crystal. M.p. = 240-241 ℃ (lit. [1] 240.9-244.8 ℃). ^{1}H NMR ($CDCl_3$, 400 MHz) δ 8.25-8.20 (m, 3 H, Ph+5-flavone), 7.67 (ddd, *J* = 8.5, 7.0, 1.6 Hz, 1 H, 7-flavone), 7.56 (d, *J* = 8,4 Hz, 1 H, 8-flavone), 7.39 (ddd, *J* = 7.8, 7.3, 0.7 Hz, 1 H, 6-flavone), 7.04 (d, *J* = 9.1 Hz, 2 H, Ph), 6.93 (br, 1 H, -OH), 3.88 (s, 3 H, -OMe). ^{13}C NMR ($CDCl_3$, 100 MHz) δ 160.45, 154.44, 145.61, 138.14, 133.48, 129.40, 124.72, 124.47, 123.58, 121.34, 118.30, 114.04, 55.36. IR (ZnSe) 3196, 1604, 1257 cm^{-1}. ESI-HRMS calcd. for $C_{16}H_{13}O_4$ (M^{+}+1) 269.0808, found 269.0804.

2-(4-(diethylamino)phenyl)-3-hydroxy-4H-chromen-4-one (N-3HF)

Yellow crystal. M.p. = 162-163 ℃ (lit. [2] 151-152 ℃). ^{1}H NMR ($CDCl_3$, 400 MHz) δ 8.21 (d, *J* = 7.4 Hz, 1 H, 5-flavone), 8.15 (d, *J* = 9.0 Hz, 2 H, Ph), 7.62 (dd, *J* = 7.2, 7.2 Hz, 1 H, 7-flavone), 7.53 (d, *J* = 8.3 Hz, 1 H, 8-flavone), 7.36 (dd, *J* = 7.3, 7.3 Hz, 1 H, 6-flavone), 6.88 (br, 1 H, -OH), 6.75 (d, *J* = 9.0, 2 H, Ph), 3.43 (q, *J* = 6.9 Hz, 4 H, -NEt_2), 1.21 (t, *J* = 6.9 Hz, 6 H, -NEt_2). ^{13}C NMR ($CDCl_3$, 100 MHz) δ 172.36, 155.06, 148.97, 146.82, 136.77, 132.71, 129.48, 125.20, 124.09, 120.92, 117.95, 117.22, 110.98, 44.4,4, 12.59. IR (ZnSe) 3279, 1597, 1200 cm^{-1}. ESI-HRMS calcd. for $C_{19}H_{20}O_3N$ (M^{+}+1) 310.1438, found 310.1438.

7-(Diethylamino)-3-hydroxy-2-(4-(trifluoromethyl)phenyl)-4H-chromen-4-one (F-NHF)

Yellow solid. M.P. = 218-219 ℃. ^{1}H NMR ($CDCl_3$, 400 MHz) δ 8.33 (d, *J* = 8.3 Hz, 2 H, Ph), 8.00 (d, *J* = 9.2 Hz, 1 H, 5-flavone), 7.72 (d, *J* = 8.4 Hz, 2 H, Ph), 7.24 (br, 1 H, -OH), 6.76 (dd, *J* = 9.2, 2.4 Hz, 1 H, 6-flavone), 6.52 (d, *J* = 2.4 Hz, 1 H, 8-flavone), 3.47 (q, *J* = 7.1 Hz, 4 H, -NEt_2), 1.25 (t, *J* = 7.1 Hz, 6 H, -NEt_2). ^{13}C NMR ($CDCl_3$, 100 MHz) δ 172.10, 158.22, 152.30, 140.84, 138.54, 135.21, 130.83, 130.50, 127.41, 126.74, 126.40, 125.31, 125.28, 111.03, 110.04, 95.90, 44.83, 12.51. IR (ZnSe) 3260, 1606, 1325, 1134 cm^{-1}. ESI-HRMS calcd. for $C_{20}H_{19}F_3O_3N_2$ (M^{+}+1) 378.1312, found 378.1311.

7-(Diethylamino)-3-hydroxy-2-phenyl-4H-chromen-4-one (H-NHF)

Brownish crystal. M.P. = 155-156 ℃ (lit. [4] 153-154 ℃). ^{1}H NMR ($CDCl_3$, 400 MHz) δ 8.21 (d, *J* = 7.6 Hz, 2 H, Ph), 8.00 (d, *J* = 9.1 Hz, 1 H, 5-flavone), 7.49 (t, *J* = 7.6 Hz, 2 H, Ph), 7.40 (t, *J* = 7.3 Hz, 1 H, Ph), 6.75 (dd, *J* = 9.1, 1.7 Hz, 1 H, 6-flavone), 6.52 (d, *J* = 1.7 Hz, 1 H, 8-flavone), 3.45 (q, *J* = 7.0 Hz, 4 H, -NEt_2), 1.23 (t, *J* = 6.9 Hz, 6 H, -NEt_2). ^{13}C NMR ($CDCl_3$, 100 MHz) δ 158.15, 152.00, 142.89, 137.67, 131.76, 129.34, 128.42, 127.32, 126.60, 110.78, 110.17, 96.00, 44.77, 12.51. IR (ZnSe) 3272, 1614, 1269 cm^{-1}. ESI-HRMS calcd. for $C_{19}H_{20}O_3N$ (M^{+}+1) 310.1438, found 310.1429.

7-(Diethylamino)-3-hydroxy-2-(4-methoxyphenyl)-4H-chromen-4-one (O-NHF)

Yellow crystal. M.P. = 127-128 ℃. ^{1}H NMR ($CDCl_3$, 400 MHz) δ 8.16 (d, J = 9.0 Hz, 2 H, Ph), 7.98 (d, J = 9.1 Hz, 1 H, 5-flavone), 7.00 (d+br, J = 9.0 Hz, 2 H, Ph+OH), 6.72 (dd, J = 9.1, 2.4 Hz, 1 H, 6-flavone), 6.50 (d, J = 2.3 Hz, 1 H, 8-flavone), 3.85 (s, 3 H, -OMe), 3.43 (q, J = 7.1 Hz, 4 H, -NEt_2), 1.22 (t, J = 7.1 Hz, 6 H, -NEt_2). ^{13}C NMR ($CDCl_3$, 100 MHz) δ 160.40, 157.93, 151.79, 143.21, 136.75, 128.95, 126.46, 124.28, 113.86, 110.58, 110.17, 96.02, 55.31, 44.72, 12.49. IR (ZnSe) 2980, 1604, 1258, 1175 cm^{-1}. ESI-HRMS calcd. for $C_{20}H_{22}O_4N$ (M^{+}+1) 340.1543, found 340.1533.

7-(Diethylamino)-2-(4-(diethylamino)phenyl)-3-hydroxy-4H-chromen-4-one (N-NHF)

Red crystal. M.P. = 157-158 ℃. ^{1}H NMR ($CDCl_3$, 400 MHz) δ 8.09 (d, J = 9.2 Hz, 2 H, Ph), 7.97 (d, J = 9.1 Hz, 1 H, 5-flavone), 6.92 (br, 1 H, -OH), 6.77-6.70 (m, 3 H, 6-flavone+Ph), 6.51 (d, J = 2.4 Hz, 1 H, 8-flavone), 3.48-3.38 (m, 8 H, -NEt_2), 1.26-1.16 (m, 12 H, -NEt_2). ^{13}C NMR ($CDCl_3$, 100 MHz) δ 157.78, 151.50, 148.42, 144.84, 135.91, 132.26, 128.93, 126.35, 118.18, 111.01, 110.44, 110.33, 96.19, 44.72, 44.42, 12.60, 12.54. IR (ZnSe) 2980, 1600, 1184 cm^{-1}. ESI-HRMS calcd. for $C_{23}H_{29}O_3N_2$ (M^{+}+1) 381.2173, found 381.2182.

3. ^{1}H NMR and ^{13}C NMR spectra of 3HFs, NHFs, and ^{19}F spectrum of F-NHF

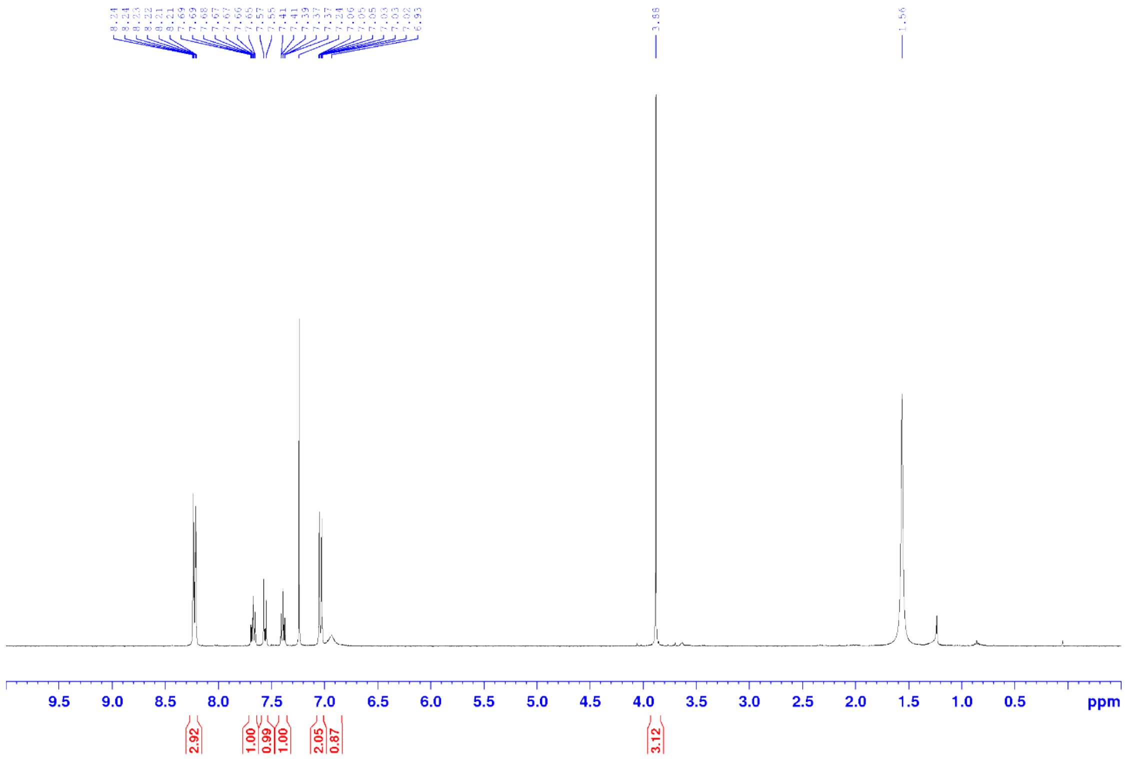

Figure S1. ^{1}H NMR of **O-3HF** in $CDCl_3$.

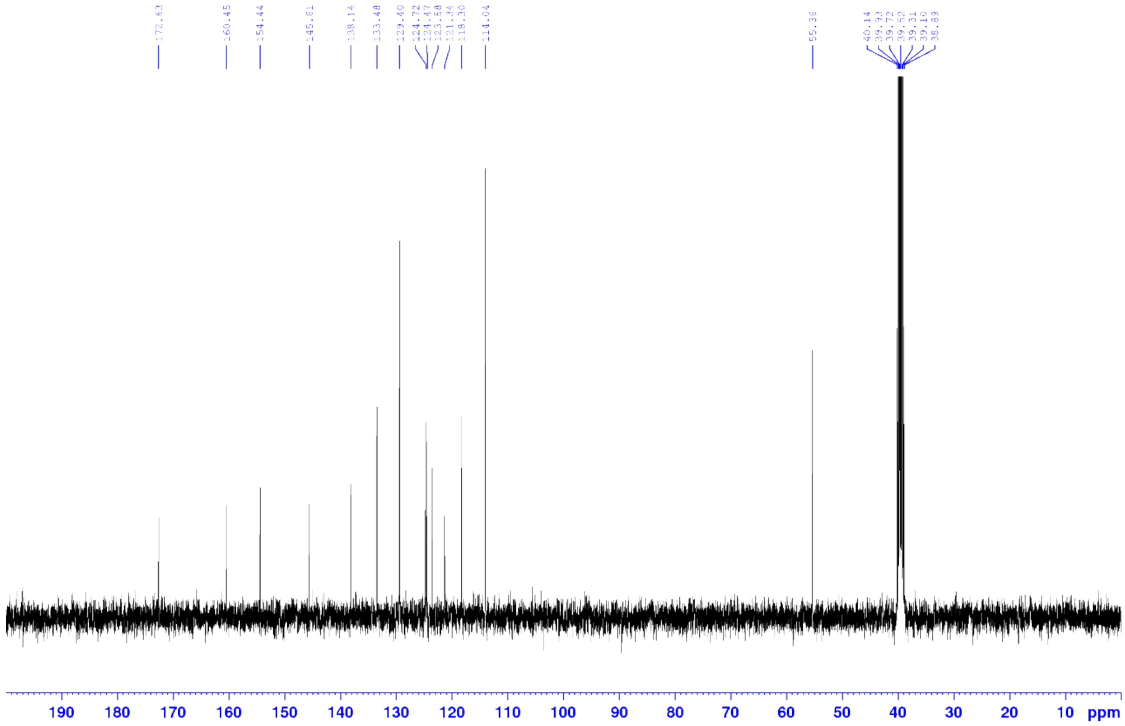

Figure S2. ^{13}C NMR of **O-3HF** in DMSO-d_6.

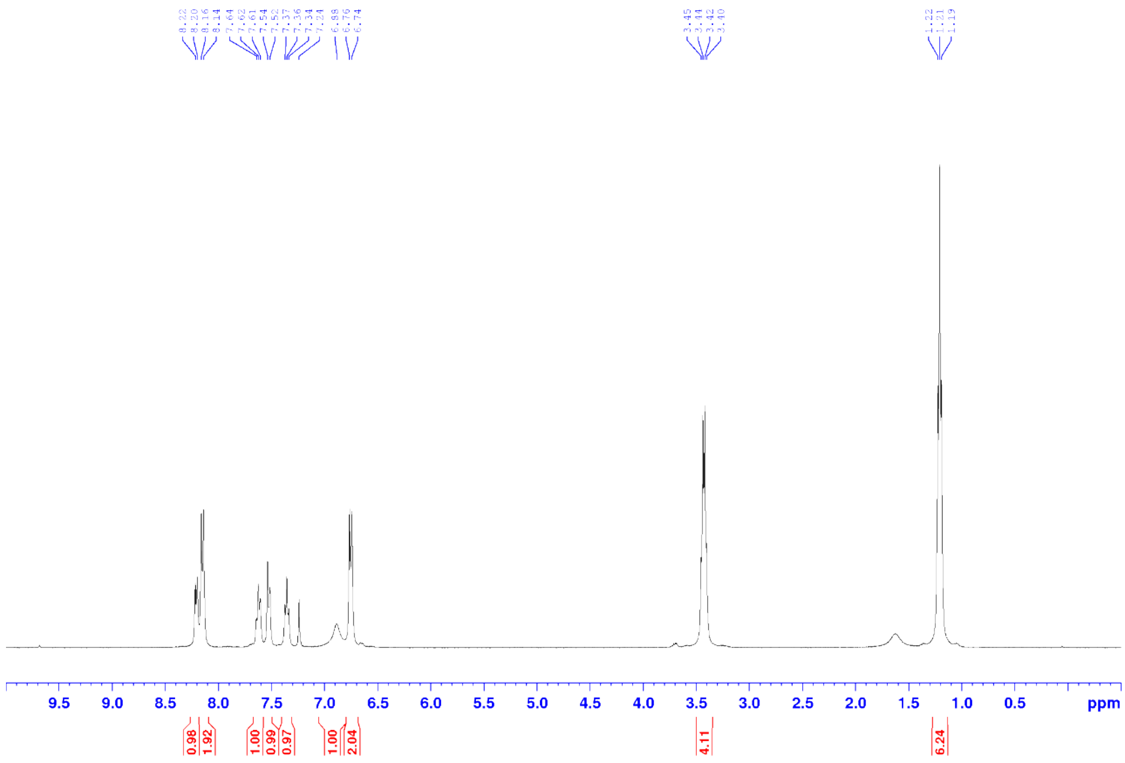

Figure S3. ^1H NMR of **N-3HF** in $CDCl_3$.

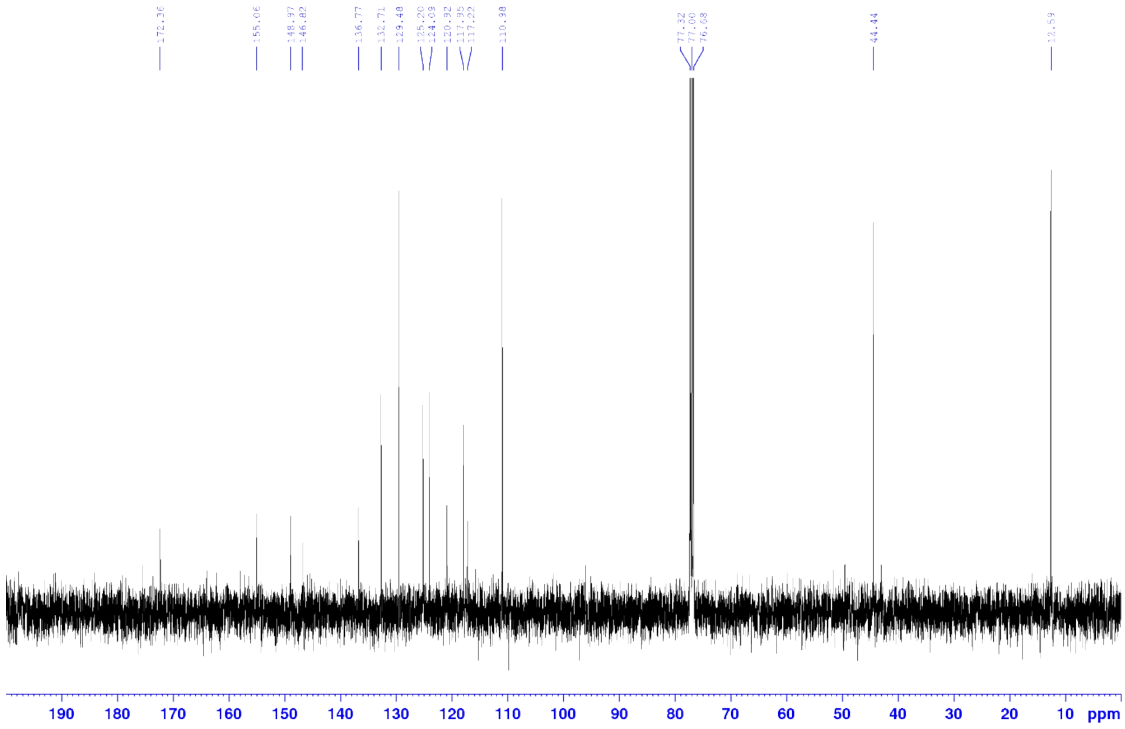

Figure S4. ^{13}C NMR of **N-3HF** in $CDCl_3$.

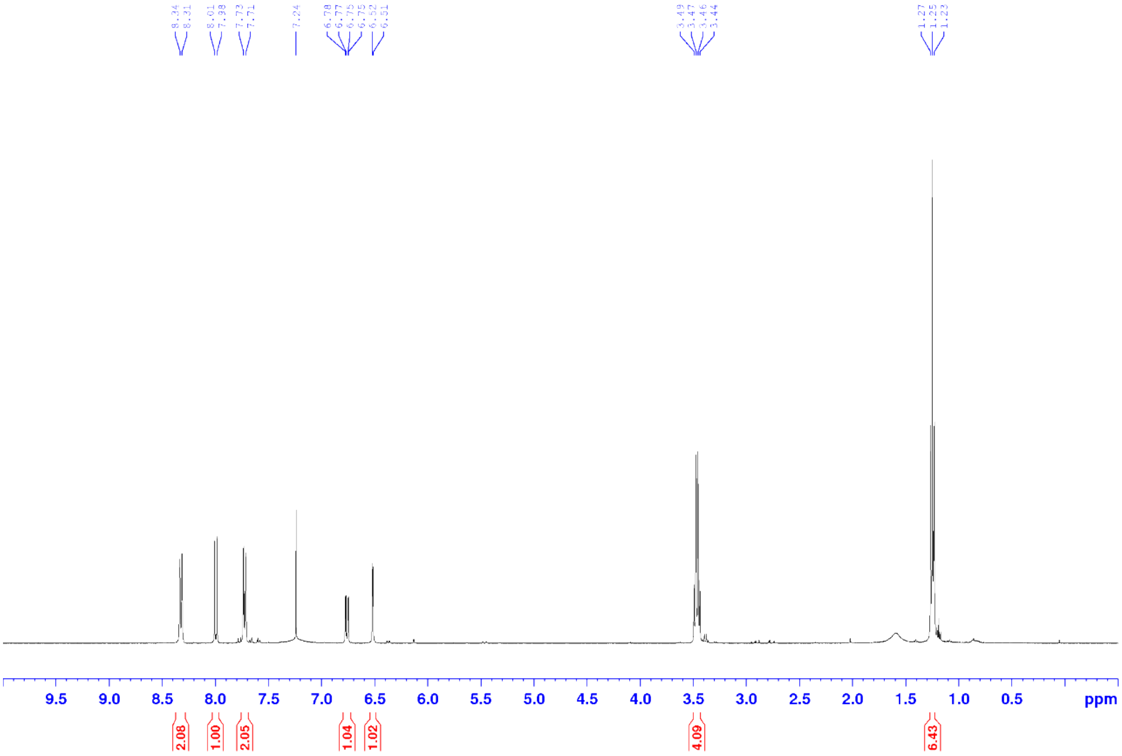

Figure S5. ¹H NMR of **F-NHF** in $CDCl_3$.

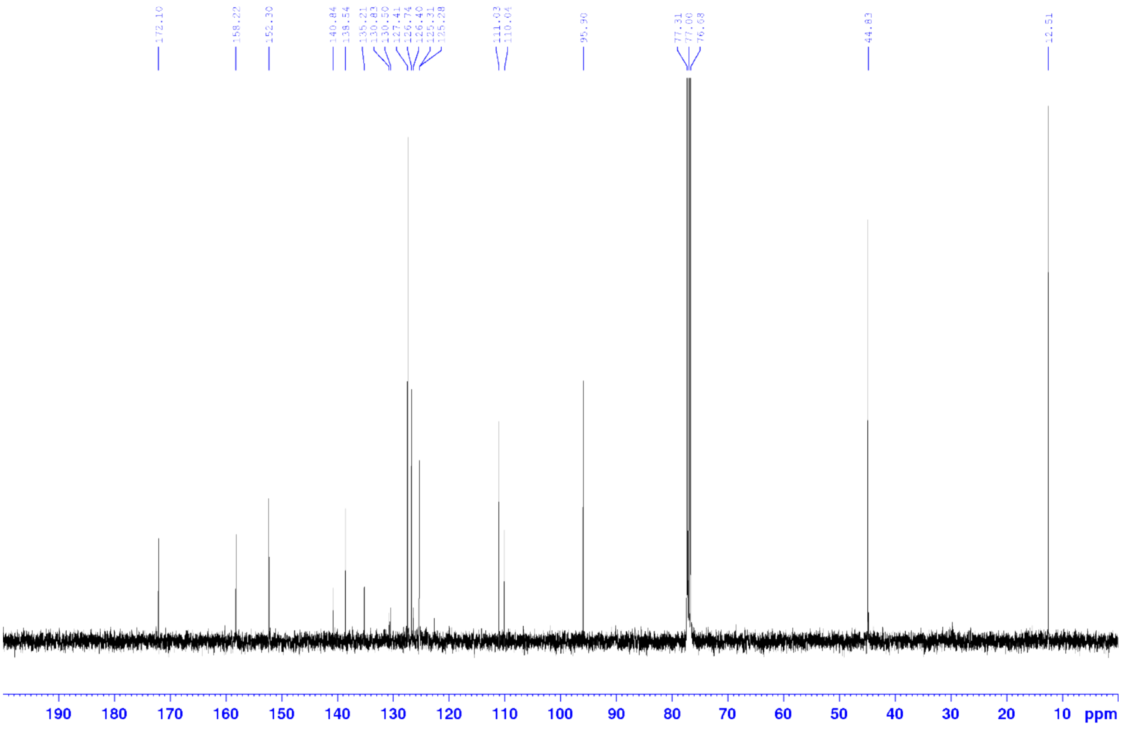

Figure S6. ¹³C NMR of **F-NHF** in $CDCl_3$.

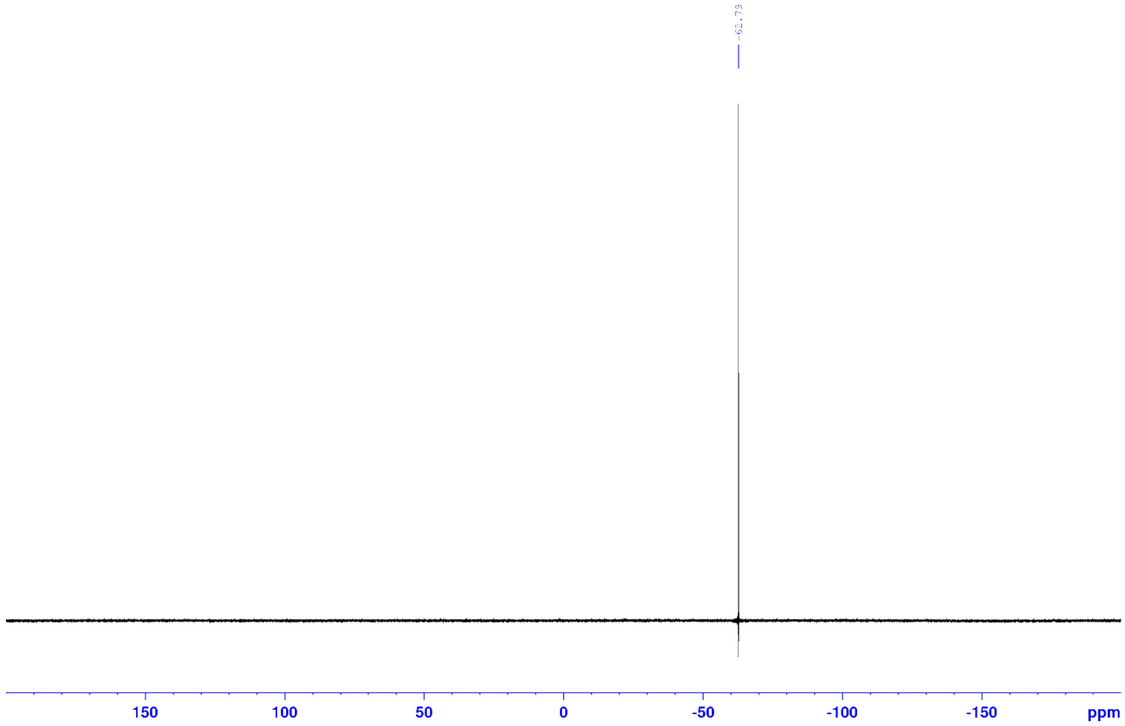

Figure S7. ^{19}F NMR of **F-NHF** in $CDCl_3$.

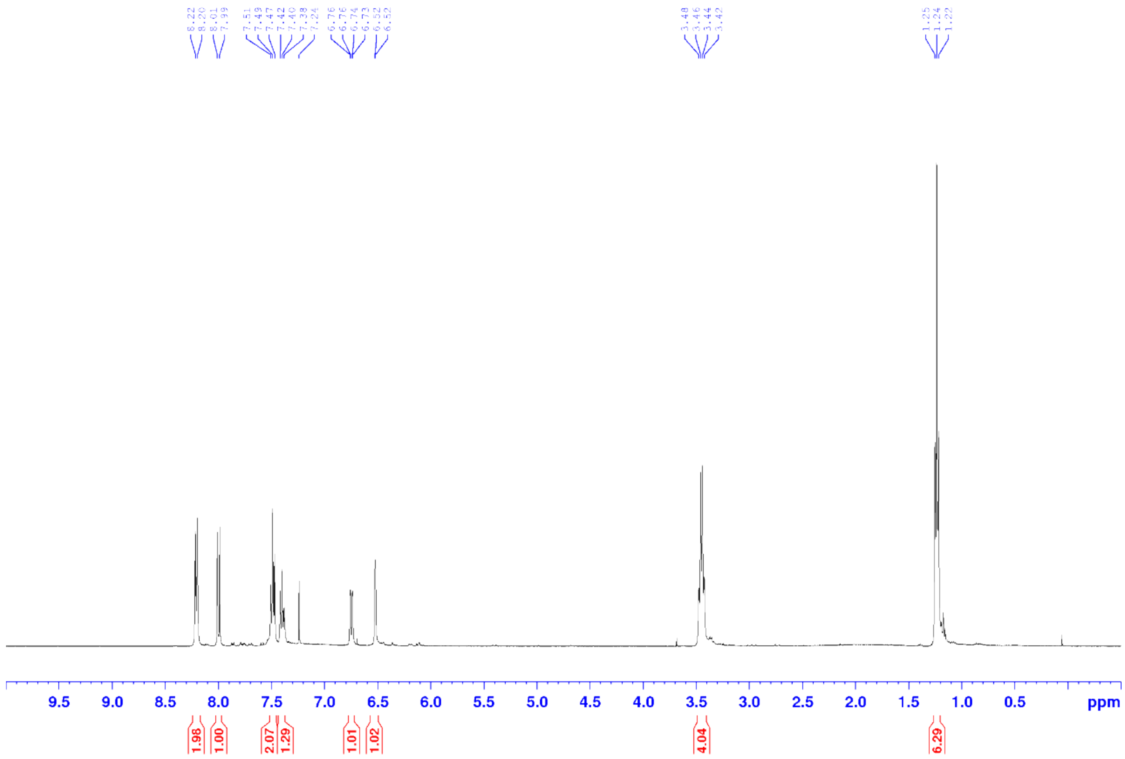

Figure S8. ^{1}H NMR of **H-NHF** in $CDCl_3$.

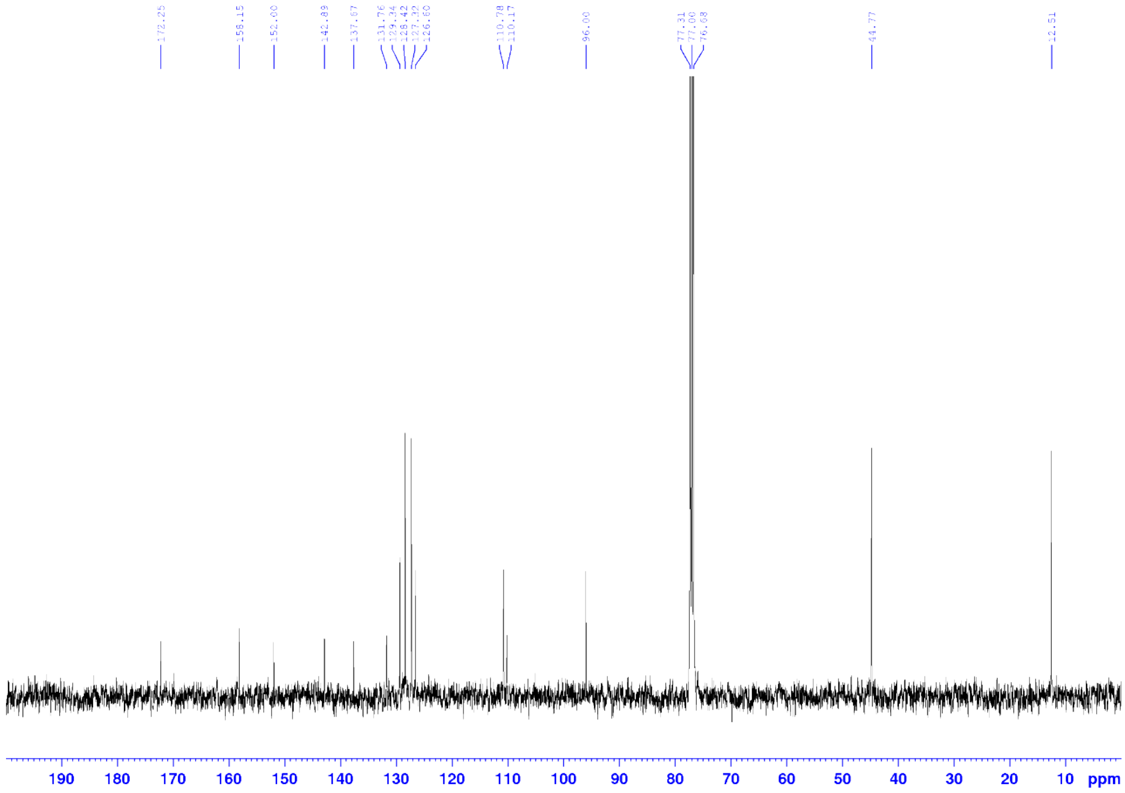

Figure S9. ^{13}C NMR of **H-NHF** in $CDCl_3$.

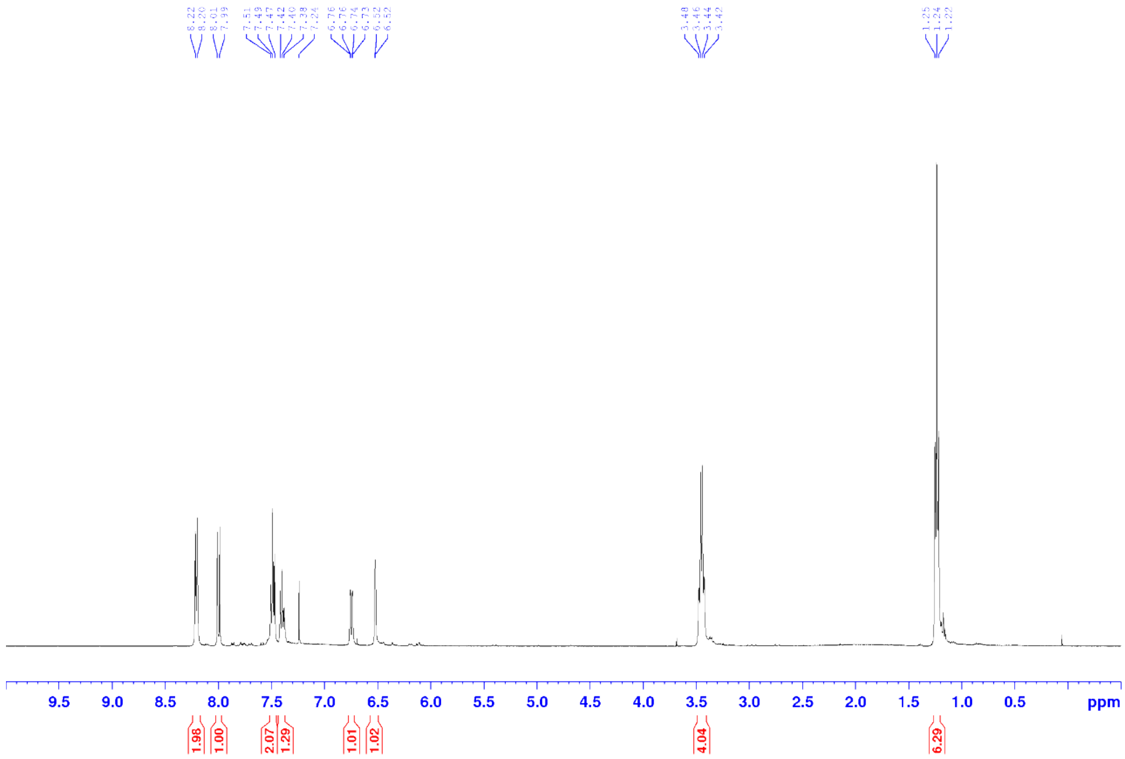

Figure S10. ^{1}H NMR of **O-NHF** in $CDCl_3$.

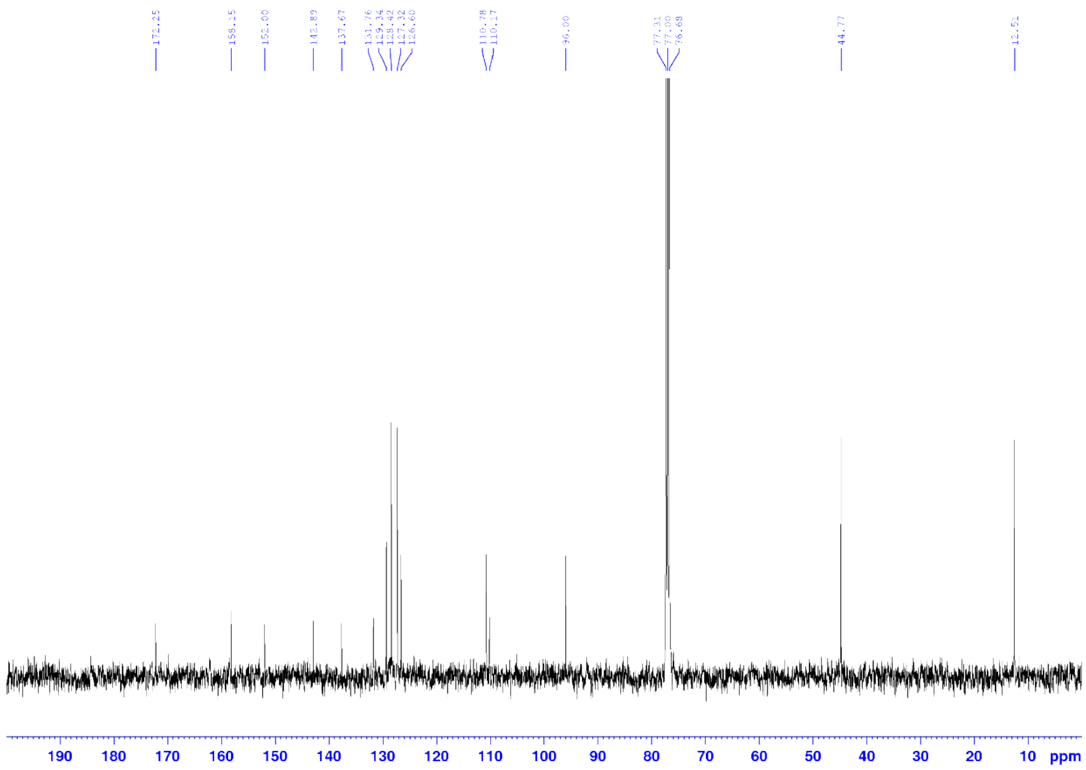

Figure S11. ^{13}C NMR of **O-NHF** in $CDCl_3$.

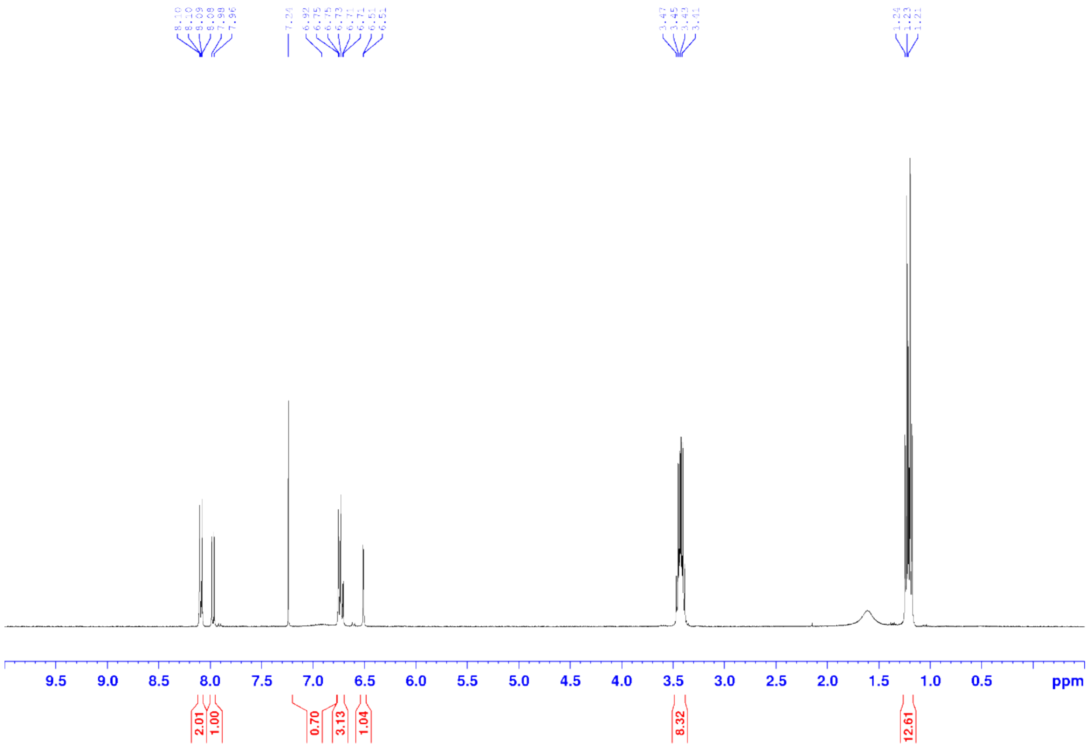

Figure S12. ^{1}H NMR of **N-NHF** in $CDCl_3$.

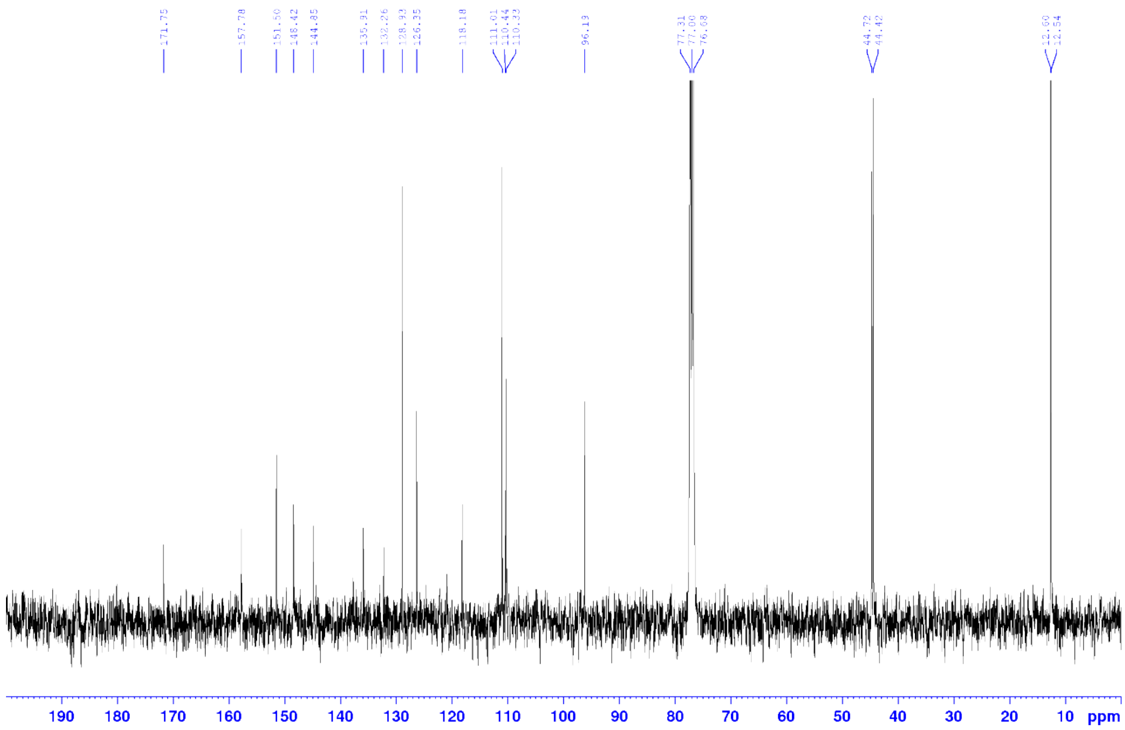

Figure S13. ^{13}C NMR of N-NHF in $CDCl_3$.

3. Spectroscopical measurement. Steady-state absorption and emission spectra were recorded by a double-beam spectrophotometer (Hitachi U-3310) and a fluorescence spectrometer (Edinburgh FS980), respectively. The nanosecond time-resolved studies were performed by a time-correlated single photon counting (TCSPC) technique (Edinburgh FLS980) with a picosecond pulsed diode laser as the excitation light source. Both excitation and emission wavelengths of FLS 980 were carefully calibrated. The samples were prepared in a 1-cm length cuvette with an absorbance of 0.3 (approximately 2×10^{-5} M) at the excitation wavelength.

The picosecond time-resolved studies were performed using a time-correlated single photon counting (TCSPC) technique, where a ~120 fs laser (400 nm) was employed as the pumping source. Incorporating this with a microchannel detector yielded a time resolution of approximately 20 ps. As for the fluorescence up-conversion measurement, a stable 120-fs LASER oscillator performed this ultrafast fluorescence up-conversion measurement (FOG100-DX, CDP corp.). A PUMP beam of 400 nm was generated by the part of the oscillator output traveling through the second harmonic generation (SHG, β-barium borate crystal). An iris selected the energy and the beam size of the PUMP after this SHG. The lens system was used to focus PUMP on the sample, to collect the fluorescence, and to focus the fluorescence on the sum-frequency BBO crystal (SHG, β-barium borate crystal), respectively. The entitled compounds are measured in a rotated cell with a transmitted collection mode. The GATE beam (800 nm) enters the delay line stage and crosses the fluorescence beam in the sum-frequency BBO with a collinear measurement. The polarized angle between PUMP and GATE is set at the magic angle (54.7˚). A monochromator was applied and coupled with a PMT to record the sum-frequency signal. In this research, the FWHM of IRF is ~150 fs.

Table S1. The computed optical excitations and molecular orbital contributions for (a) **NTF**s and (b) **NHF**s, (c) **3TF**s, and (d) **3HF**s.

(a)

Compound	Structure	State	E (eV)	Wavelength (nm)	f	Contribution	weight
H-NTF	Normal	S_1	3.20	387.3	0.2805	HOMO→LUMO	98%
	@S_0-opt	S_2	3.65	339.8	0.1462	HOMO-1→LUMO	95%
	Normal	S_1	2.62	473.4	0.2612	HOMO→LUMO	99%
	@S_1-opt	S_2	3.13	396.0	0.1714	HOMO-1→LUMO	98%
	Tautomer	S_1	2.11	586.4	0.1768	HOMO→LUMO	99%
	@S_0-opt	S_2	2.48	499.6	0.0012	HOMO-1→LUMO	99%
	Tautomer	S_1	1.57	787.3	0.0879	HOMO→LUMO	93%
	@S_1-opt	S_2	1.81	683.5	0.0151	HOMO-1→LUMO	93%
F-NTF	Normal	S_1	3.04	408.0	0.2527	HOMO→LUMO	98%
	@S_0-opt	S_2	3.48	356.0	0.1401	HOMO-1→LUMO	96%
	Normal	S_1	2.44	508.1	0.2192	HOMO→LUMO	99%
	@S_1-opt	S_2	3.00	413.9	0.1942	HOMO-1→LUMO	98%
	Tautomer	S_1	2.08	595.6	0.1938	HOMO→LUMO	99%
	@S_0-opt	S_2	2.43	509.2	0.0015	HOMO-1→LUMO	99%
	Tautomer	S_1	1.53	810.9	0.0911	HOMO→LUMO	92%
	@S_1-opt	S_2	1.76	703.0	0.0158	HOMO-1→LUMO	92%
O-NTF	Normal	S_1	3.26	380.7	0.3941	HOMO→LUMO	99%
	@S_0-opt	S_2	3.72	333.7	0.1578	HOMO-1→LUMO	94%
	Normal	S_1	2.72	456.2	0.4323	HOMO→LUMO	99%
	@S_1-opt	S_2	3.29	376.3	0.1593	HOMO-1→LUMO	97%
	Tautomer	S_1	2.12	584.0	0.2332	HOMO→LUMO	99%
	@S_0-opt	S_2	2.52	491.5	0.0008	HOMO-1→LUMO	99%
	Tautomer	S_1	1.66	747.6	0.1308	HOMO→LUMO	96%
	@S_1-opt	S_2	1.91	647.9	0.0083	HOMO-1→LUMO	96%
N-NTF	Normal	S_1	3.07	403.4	0.7029	HOMO→LUMO	98%
	@S_0-opt	S_2	3.68	337.0	0.0984	HOMO-1→LUMO	95%
	Normal	S_1	2.71	457.8	0.7836	HOMO→LUMO	98%
	@S_1-opt	S_2	3.44	359.0	0.12171	HOMO-1→LUMO	94%
	Tautomer	S_1	2.11	588.6	0.41	HOMO→LUMO	98%
	@S_0-opt	S_2	2.56	484.1	0.0008	HOMO-1→LUMO	99%
	Tautomer	S_1	1.72	722.2	0.2396	HOMO→LUMO	97%
	@S_1-opt	S_2	2.03	609.7	0.0082	HOMO-1→LUMO	98%

(b)

Compound	Structure	State	E (eV)	Wavelength (nm)	f	Contribution	weight
H-NHF	Normal	S_1	3.17	391.4	0.4986	HOMO→LUMO	99%
	@S_0-opt	S_2	3.79	327.2	0.194	HOMO-1→LUMO	91%
	Normal	S_1	2.80	443.2	0.4305	HOMO→LUMO	99%
	@S_1-opt	S_2	3.52	352.7	0.27	HOMO-1→LUMO	95%
	Tautomer	S_1	2.43	511.2	0.5525	HOMO→LUMO	100%
	@S_0-opt	S_2	3.52	352.0	0.0004	HOMO-2→LUMO	99%
	Tautomer	S_1	2.16	574.2	0.5311	HOMO→LUMO	101%
	@S_1-opt	S_2	3.14	395.0	0.0000	HOMO-2→LUMO	99%
F-NHF	Normal	S_1	3.04	407.8	0.4513	HOMO→LUMO	99%
	@S_0-opt	S_2	3.68	336.6	0.1997	HOMO-1→LUMO	92%
	Normal	S_1	2.60	476.2	0.3508	HOMO→LUMO	99%
	@S_1-opt	S_2	3.39	366.2	0.2528	HOMO-1→LUMO	95%
	Tautomer	S_1	2.37	522.3	0.5724	HOMO→LUMO	100%
	@S_0-opt	S_2	3.50	354.2	0.0011	HOMO-2→LUMO	98%
	Tautomer	S_1	2.10	589.1	0.5439	HOMO→LUMO	101%
	@S_1-opt	S_2	3.10	399.6	0	HOMO-2→LUMO	99%
O-NHF	Normal	S_1	3.18	390.5	0.6582	HOMO→LUMO	99%
	@S_0-opt	S_2	3.78	328.0	0.2216	HOMO-1→LUMO	93%
	Normal	S_1	2.89	429.0	0.6999	HOMO→LUMO	98%
	@S_1-opt	S_2	3.55	349.4	0.2503	HOMO-1→LUMO	93%
	Tautomer	S_1	2.39	519.0	0.6448	HOMO→LUMO	100%
	@S_0-opt	S_2	3.56	348.5	0.0006	HOMO-3→LUMO	98%
	Tautomer	S_1	2.15	577.4	0.6264	HOMO→LUMO	101%
	@S_1-opt	S_2	3.21	386.3	0.0001	HOMO-2→LUMO	98%
N-NHF	Normal	S_1	2.99	415.1	0.9709	HOMO→LUMO	98%
	@S_0-opt	S_2	3.69	336.2	0.1179	HOMO-1→LUMO	95%
	Normal	S_1	2.77	448.1	1.0253	HOMO→LUMO	98%
	@S_1-opt	S_2	3.58	346.4	0.1132	HOMO-1→LUMO	94%
	Tautomer	S_1	2.28	544.1	0.8837	HOMO→LUMO	98%
	@S_0-opt	S_2	3.35	370.2	0.0888	HOMO-1→LUMO	99%
	Tautomer	S_1	2.10	591.0	0.8629	HOMO→LUMO	97%
	@S_1-opt	S_2	3.26	380.3	0.1309	HOMO-1→LUMO	98%

(c)

Compound	Structure	State	E (eV)	Wavelength (nm)	f	Contribution	weight
H-3TF	Normal @S_0-opt	S_1	3.34	371.4	0.2099	HOMO→LUMO	98%
		S_2	3.94	315.0	0.0067	HOMO-3→LUMO	24%
						HOMO-2→LUMO	55%
						HOMO-1→LUMO	10%
	Normal @S_1-opt	S_1	2.76	450.0	0.2652	HOMO→LUMO	99%
		S_2	3.73	332.6	0.0195	HOMO-3→LUMO	13%
						HOMO-2→LUMO	60%
						HOMO-1→LUMO	15%
	Tautomer @S_0-opt	S_1	2.03	611.0	0.1273	HOMO→LUMO	99%
		S_2	2.30	538.3	0.0013	HOMO-1→LUMO	99%
	Tautomer @S_1-opt	S_1	1.41	877.8	0.0449	HOMO-1→LUMO	16%
						HOMO→LUMO	84%
		S_2	1.62	763.4	0.0256	HOMO-1→LUMO	84%
						HOMO→LUMO	16%
F-3TF	Normal @S_0-opt	S_1	3.26	380.7	0.2076	HOMO→LUMO	98%
		S_2	3.89	318.5	0.0068	HOMO-2→LUMO	85%
	Normal @S_1-opt	S_1	2.65	468.4	0.2483	HOMO→LUMO	99%
		S_2	3.63	341.6	0.0187	HOMO-2→LUMO	81%
	Tautomer @S_0-opt	S_1	1.99	623.0	0.1308	HOMO→LUMO	99%
		S_2	2.26	549.0	0.0016	HOMO-1→LUMO	99%
	Tautomer @S_1-opt	S_1	1.34	927.0	0.0412	HOMO-1→LUMO	18%
						HOMO→LUMO	82%
		S_2	1.55	799.9	0.0285	HOMO-1→LUMO	82%
						HOMO→LUMO	18%
O-3TF	Normal @S_0-opt	S_1	3.29	376.3	0.3379	HOMO→LUMO	98%
		S_2	3.92	316.2	0.0525	HOMO-3→LUMO	32%
						HOMO-2→LUMO	19%
						HOMO-1→LUMO	42%
	Normal @S_1-opt	S_1	2.81	440.5	0.4097	HOMO→LUMO	99%
		S_2	3.76	330.0	0.1093	HOMO-2→LUMO	37%
						HOMO-1→LUMO	48%
	Tautomer @S_0-opt	S_1	2.09	591.9	0.2005	HOMO→LUMO	99%
		S_2	2.36	525.3	0	HOMO-1→LUMO	99%
	Tautomer @S_1-opt	S_1	1.53	811.5	0.075	HOMO-1→LUMO	11%
						HOMO→LUMO	88%
		S_2	1.73	718.6	0.0215	HOMO-1→LUMO	88%
						HOMO→LUMO	11%
N-3TF	Normal @S_0-opt	S_1	2.99	415.0	0.6201	HOMO→LUMO	99%
		S_2	3.69	336.3	0.0344	HOMO-1→LUMO	96%

Normal	S_1	2.63	471.5	0.6251	HOMO→LUMO	99%
@S_1-opt	S_2	3.44	360.1	0.0443	HOMO-1→LUMO	97%
Tautomer	S_1	2.07	599.8	0.3379	HOMO→LUMO	97%
@S_0-opt	S_2	2.40	516.2	0.0009	HOMO-1→LUMO	99%
Tautomer	S_1	1.65	751.6	0.1568	HOMO→LUMO	92%
@S_1-opt	S_2	1.87	661.5	0.0186	HOMO-1→LUMO	94%

(d)

Compound	Structure	State	E (eV)	Wavelength (nm)	f	Contribution	weight
H-3HF	Normal @S_0-opt	S_1	3.48	356.5	0.5051	HOMO→LUMO	98%
		S_2	4.06	305.7	0.0028	HOMO-4→LUMO	13%
						HOMO-3→LUMO	81%
	Normal @S_1-opt	S_1	3.04	407.3	0.5656	HOMO→LUMO	99%
		S_2	4.01	309.0	0.1413	HOMO-1→LUMO	91%
	Tautomer @S_0-opt	S_1	2.52	492.2	0.4712	HOMO→LUMO	101%
		S_2	3.35	370.6	0	HOMO-1→LUMO	99%
	Tautomer @S_1-opt	S_1	2.26	549.1	0.4504	HOMO→LUMO	101%
		S_2	2.96	418.2	0	HOMO-1→LUMO	99%
F-3HF	Normal @S_0-opt	S_1	3.46	358.7	0.4921	HOMO→LUMO	98%
		S_2	4.00	310.4	0.0005	HOMO-4→LUMO	95%
	Normal @S_1-opt	S_1	3.03	408.9	0.575	HOMO→LUMO	99%
		S_2	3.94	314.9	0	HOMO-4→LUMO	96%
	Tautomer @S_0-opt	S_1	2.50	496.2	0.4795	HOMO→LUMO	101%
		S_2	3.31	374.4	0	HOMO-1→LUMO	99%
	Tautomer @S_1-opt	S_1	2.22	558.5	0.4541	HOMO→LUMO	101%
		S_2	2.90	427.3	0	HOMO-1→LUMO	99%
O-3HF	Normal @S_0-opt	S_1	3.32	373.7	0.6874	HOMO→LUMO	99%
		S_2	4.05	306.1	0.1271	HOMO-1→LUMO	93%
	Normal @S_1-opt	S_1	2.95	420.6	0.7005	HOMO→LUMO	99%
		S_2	3.93	315.3	0.0976	HOMO-1→LUMO	92%
	Tautomer @S_0-opt	S_1	2.46	503.3	0.5965	HOMO→LUMO	101%
		S_2	3.39	366.2	0	HOMO-2→LUMO	99%
	Tautomer @S_1-opt	S_1	2.25	552.0	0.5773	HOMO→LUMO	101%
		S_2	3.07	403.4	0	HOMO-2→LUMO	99%
N-3HF	Normal @S_0-opt	S_1	2.92	424.0	0.8462	HOMO→LUMO	99%
		S_2	3.92	316.4	0.0367	HOMO-1→LUMO	88%
	Normal @S_1-opt	S_1	2.68	462.6	0.7782	HOMO→LUMO	99%
		S_2	3.7799	328.0	0.0202	HOMO-1→LUMO	94%
	Tautomer @S_0-opt	S_1	2.33	531.3	0.8704	HOMO→LUMO	101%
		S_2	3.29	376.5	0.1113	HOMO-1→LUMO	97%
	Tautomer @S_1-opt	S_1	2.19	566.4	0.8527	HOMO→LUMO	101%
		S_2	3.21	386.4	0.1429	HOMO-1→LUMO	92%

Table S2. The energy difference between the S1(N) and S1(T) states (ΔE_R^{ES}) and energy differences between substituted and unsubstituted systems ($\Delta(\Delta E_R^{ES})$) in (a) **NTF**s and **NHF**s, (b) **3TF**s and **3HF**s.

(a)

compound	F-NTF	H-NTF	O-NTF	N-NTF	F-NHF	H-NHF	O-NHF	N-NHF
ΔE_R^{ES} (eV)	0.781	0.785	0.932	1.770	0.184	0.154	0.330	0.246
$\Delta(\Delta E_R^{ES})$ (eV)	-0.004	0.000	0.147	0.985	0.030	0.000	0.176	0.092

(b)

compound	F-3TF	H-3TF	O-3TF	N-3TF	F-3HF	H-3HF	O-3HF	N-3HF
ΔE_R^{ES} (eV)	1.026	1.050	1.015	0.782	0.421	0.394	0.309	0.110
$\Delta(\Delta E_R^{ES})$ (eV)	-0.024	0.000	-0.035	-0.268	0.027	0.000	-0.085	-0.284

Table S3. Calculated vibrational frequencies and bond lengths of C=O and X-H (X = S or O) in S_0 and S_1-optimized normal form structures of **NTF**s and **NHF**s, along with experimental C=O bond lengths obtained from single-crystal XRD and proton transfer rates.

Bond\Structure	F-NTF	H-NTF	O-NTF	N-NTF	F-NHF	H-NHF	O-NHF	N-NHF
vibrational frequency @S_0-opt	2581.49	2585.62	2587.08	2585.45	3518.05	3525.75	3536.06	3543.08
vibrational frequency @S_1-opt	2568.81	2504.17	2315.83	2339.73	3555.85	3484.82	3348.89	3253.57
S-H @S_0-opt (Å)	1.352	1.352	1.351	1.351	0.980	0.980	0.979	0.978
S-H @S_1-opt (Å)	1.353	1.359	1.379	1.376	0.978	0.982	0.990	0.996
Δ (S-H)^a (Å)	0.001	0.008	0.028	0.025	-0.003	0.002	0.011	0.017
C=O @S_0-opt (Å)	1.233	1.234	1.234	1.236	1.241	1.242	1.243	1.244
C=O @S_1-opt (Å)	1.248	1.256	1.264	1.270	1.257	1.265	1.270	1.272
Δ (C=O)^a (Å)	0.015	0.022	0.030	0.034	0.016	0.023	0.027	0.028
C=O @Exp (Å)	1.237	1.240	1.238	1.244	--	--	--	--
k_{PT} (fs)	398	232	123	101				

Table S4. Calculated vibrational frequencies and bond lengths of C=O and X-H (X = S or O) in S_0 and S_1-optimized normal form structures of **3TF**s and **3HF**s..

Bond\Structure	F-3TF	H-3TF	O-3TF	N-3TF	F-3HF	H-3HF	O-3HF	N-3HF
vibrational frequency @S_0-opt	2600.12	2604.02	2600.78	2597.09	3550.59	3560.05	3556.97	3561.23
vibrational frequency @S1-opt	2370.33	2338.86	2560.12	2085.94	3229.2313	3176.17	3185.23	3275.61
S-H @S0-opt (Å)	1.35025	1.34991	1.35017	1.35045	0.97836	0.97771	0.97779	0.97749
S-H @S1-opt (Å)	1.37592	1.37592	1.3454	1.40824	0.99849	1.00159	1.00053	0.99436
D (S-H)a (Å)	0.02567	0.02601	-0.00477	0.05779	0.02013	0.02388	0.02274	0.01687
C=O @S0-opt (Å)	1.22853	1.22965	1.23087	1.23295	1.23562	1.23723	1.23893	1.24136
C=O @S1-opt (Å)	1.2495	1.25688	1.25699	1.2645	1.25575	1.25816	1.26023	1.26222
D (C=O)a (Å)	0.02097	0.02723	0.02612	0.03155	0.02013	0.02093	0.0213	0.02086

Table S5. Charges on the donor (S or O) and acceptor (O) atoms, and the corresponding charge differences (Δq) between them in the S_1 state, obtained from S_1(N)-optimized geometries. (a) **NTF**s and **NHF**s; (b) **3TF**s and **3HF**s.

(a)

compound	F-NTF	H-NTF	O-NTF	N-NTF	F-NHF	H-NHF	O-NHF	N-NHF
q_{donor}	0.04	0.08	0.15	0.11	-0.69	-0.68	-0.66	-0.67
$q_{acceptor}$	-0.68	-0.71	-0.72	-0.73	-0.71	-0.74	-0.75	-0.77
Δq	0.72	0.79	0.87	0.84	0.02	0.06	0.09	0.1

(b)

compound	F-3TF	H-3TF	O-3TF	N-3TF	F-3HF	H-3HF	O-3HF	N-3HF
q_{donor}	0.35	0.35	0.31	0.12	-0.59	-0.60	-0.62	-0.67
$q_{acceptor}$	-0.67	-0.68	-0.70	-0.70	-0.68	-0.69	-0.70	-0.72
Δq	1.02	1.03	1.01	0.82	0.09	0.09	0.08	0.05

Table S6. The computed optical excitations and molecular orbital contributions with CAM-B3LYP functionals for (a) **NTF**s and (b) **NHF**s, (c) **3TF**s, and (d) **3HF**s.

(a)

Compound	State	E (eV)	Wavelength (nm)	f	Contribution	weight
H-NTF	S_1	2.84	436.8	0.3447	HOMO→LUMO	92%
	S_2	3.75	330.4	0.3141	HOMO-1→LUMO	87%
F-NTF	S_1	2.82	440.4	0.3514	HOMO→LUMO	89%
	S_2	3.65	339.8	0.2983	HOMO-1→LUMO	84%
O-NTF	S_1	2.80	442.9	0.4647	HOMO→LUMO	95%
	S_2	3.85	322.2	0.3328	HOMO-1→LUMO	87%
N-NTF	S_1	2.82	440.0	0.8097	HOMO→LUMO	96%
	S_2	4.02	308.8	0.3523	HOMO-2→LUMO	11%
					HOMO-1→LUMO	77%

(b)

Compound	State	E (eV)	Wavelength (nm)	f	Contribution	weight
H-NHF	S_1	3.22	385.0	0.3447	HOMO→LUMO	95%
	S_2	4.06	305.3	0.3078	HOMO-1→LUMO	85%
F-NHF	S_1	3.1617	392.14	0.7133	HOMO→LUMO	92%
	S_2	3.955	313.49	0.3142	HOMO-1→LUMO	87%
O-NHF	S_1	3.1914	388.5	0.878	HOMO→LUMO	96%
	S_2	4.1434	299.23	0.3234	HOMO-1→LUMO	82%
N-NHF	S_1	3.0447	407.22	1.1825	HOMO→LUMO	95%
	S_2	4.1975	295.38	0.3089	HOMO-1→LUMO	76%

(c)

Compound	State	E (eV)	Wavelength (nm)	f	Contribution	weight
H-3TF	S_1	2.81	440.6	0.3177	HOMO→LUMO	97%
	S_2	4.07	304.3	0.0223	HOMO-4→LUMO	31%
					HOMO-2→LUMO	30%
F-3TF	S_1	2.80	440.4	0.3514	HOMO→LUMO	98%
	S_2	4.04	306.9	0.0189	HOMO-3→LUMO	44%
					HOMO-2→LUMO	21%
O-3TF	S_1	2.77	447.4	0.4506	HOMO→LUMO	97%
	S_2	4.05	305.9	0.0797	HOMO-4→LUMO	23%
					HOMO-2→LUMO	16%
					HOMO-1→LUMO	28%
					HOMO→LUMO+1	13%
N-3TF	S_1	2.85	434.6	0.8138	HOMO→LUMO	95%
	S_2	4.04	307.0	0.2029	HOMO-1→LUMO	76%

(d)

Compound	State	E (eV)	Wavelength (nm)	f	Contribution	weight
	S_1	3.30	375.2	0.6752	HOMO→LUMO	98%
H-3HF	S_2	4.39	282.1	0.1042	HOMO-1→LUMO	71%
					HOMO→LUMO+1	19%
	S_1	3.28	377.8	0.6861	HOMO→LUMO	98%
F-3HF	S_2	4.37	283.7	0.1385	HOMO-1→LUMO	72%
					HOMO→LUMO+1	18%
	S_1	3.24	383.1	0.8519	HOMO→LUMO	97%
O-3HF	S_2	4.35	284.7	0.1162	HOMO-1→LUMO	69%
					HOMO→LUMO+1	19%
	S_1	3.05	406.8	1.1341	HOMO→LUMO	94%
N-3HF	S_2	4.27	290.6	0.0857	HOMO-1→LUMO	65%
					HOMO→LUMO+2	20%

Table S7. Charges on the donor (S or O) and acceptor (O) atoms, and the corresponding charge differences (Δq) between them in the S_1 state, obtained from S_1(N)-optimized geometries with CAM-B3LYP functionals. (a) **NTF**s and **NHF**s; (b) **3TF**s and **3HF**s.

(a)

compound	F-NTF	H-NTF	O-NTF	N-NTF	F-NHF	H-NHF	O-NHF	N-NHF
q_{donor}	0.24	0.23	0.20	0.14	-0.65	-0.65	-.0.65	-0.67
$q_{acceptor}$	-0.71	-0.72	-0.72	-0.72	-0.76	-0.77	-0.77	-0.80
Δq	0.95	0.95	0.92	0.86	0.11	0.12	0.12	0.12

(b)

compound	F-3TF	H-3TF	O-3TF	N-3TF	F-3HF	H-3HF	O-3HF	N-3HF
q_{donor}	0.33	0.30	0.26	0.17	-0.60	-0.61	-0.63	-0.66
$q_{acceptor}$	-0.67	-0.68	-0.69	-0.71	-0.70	-0.71	-0.73	-0.75
Δq	1.00	0.98	0.95	0.88	0.10	0.11	0.10	0.08

Table S8. Charges on the donor (S or O) and acceptor (O) atoms, and the corresponding charge differences (Δq) between them in the S_1 state, obtained from S_1(N)-optimized geometries with ωB97XD functionals for **3TF**s.

compound	F-3TF	H-3TF	O-3TF	N-3TF
q_{donor}	0.33	0.30	0.26	0.18
$q_{acceptor}$	-0.66	-0.67	-0.69	-0.70
Δq	0.99	0.98	0.95	0.88

Table S9. The computed optical excitations and molecular orbital contributions with ωB97XD functionals for **3TF**s.

Compound	State	E (eV)	Wavelength (nm)	f	Contribution	weight
H-3TF	S_1	2.76	449.1	0.3006	HOMO→LUMO	97%
	S_2	4.05	305.8	0.0227	HOMO-4→LUMO	21%
					HOMO-2→LUMO	45%
F-3TF	S_1	2.77	447.9	0.2992	HOMO→LUMO	98%
	S_2	4.03	307.8	0.0187	HOMO-3→LUMO	44%
					HOMO-2→LUMO	21%
O-3TF	S_1	2.69	460.4	0.4184	HOMO→LUMO	96%
	S_2	4.03	307.8	0.0746	HOMO-4→LUMO	22%
					HOMO-2→LUMO	27%
					HOMO-1→LUMO	22%
N-3TF	S_1	2.74	451.9	0.7281	HOMO→LUMO	94%
	S_2	4.01	309.1	0.2255	HOMO-1→LUMO	64%
					HOMO-3→LUMO	14%

Table S10. The cSAR(R'') value calculated with different functionals for **3TF**s.

Funtionals	F-3TF	H-3TF	O-3TF	N-3TF
B3LYP	0.22	0.51	0.64	0.86
CAM-B3LYP	0.24	0.32	0.59	0.73
ωB97XD	-0.64	0.32	0.59	0.72

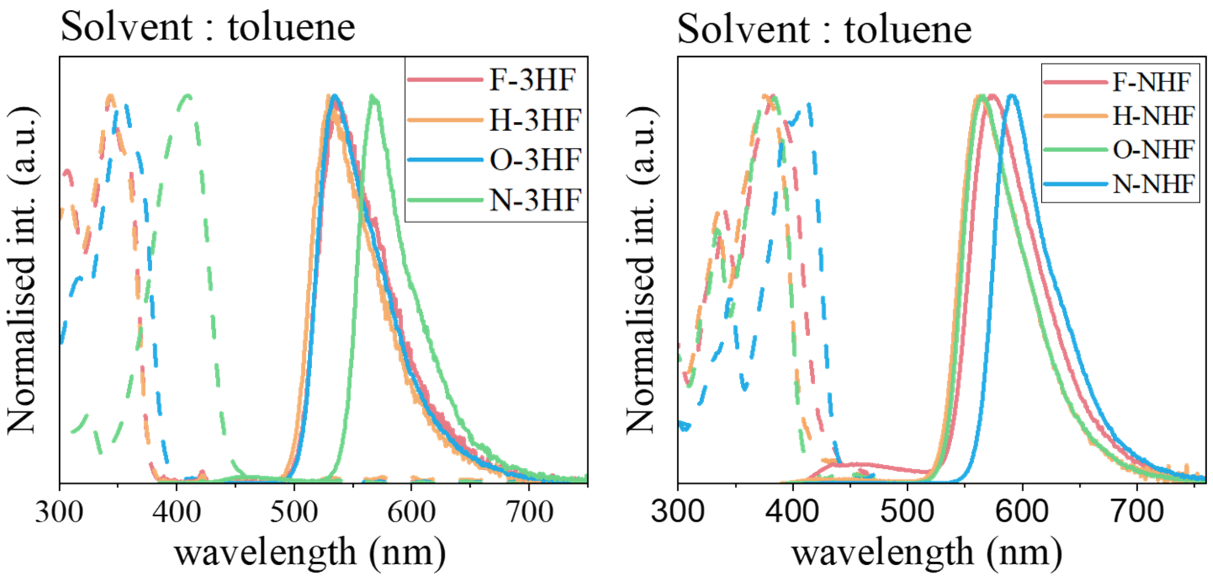

Figure S14. Steady spectra of **3HF**s (upper panel) and **NHF**s (lower panel). In the steady-state spectra, dashed lines represent absorption, while solid lines indicate emission. All measurements were performed in dilute toluene, with an excitation wavelength of the first absorption band.

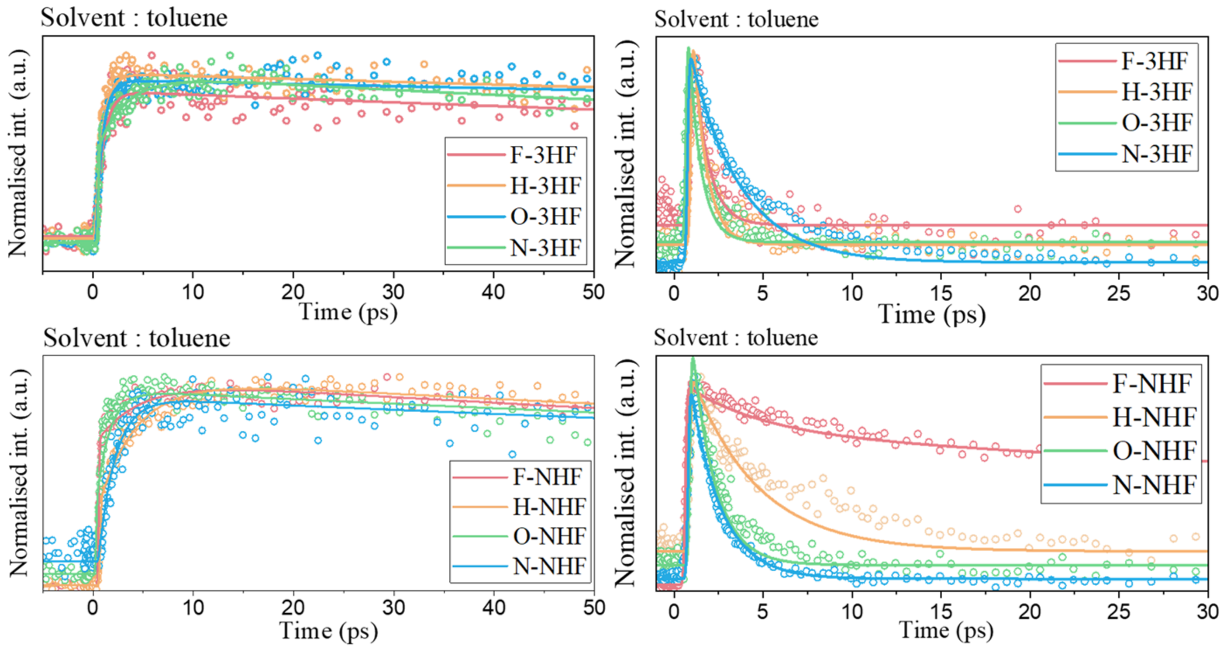

Figure S15. Kinetic spectra of **3HF**s (upper panel) and **NHF**s (lower panel). Kinetic spectra were obtained using the upconversion technique, monitored at the tautomer emission peak for each compound. All measurements were performed in dilute toluene, with an excitation wavelength of the first absorption band.

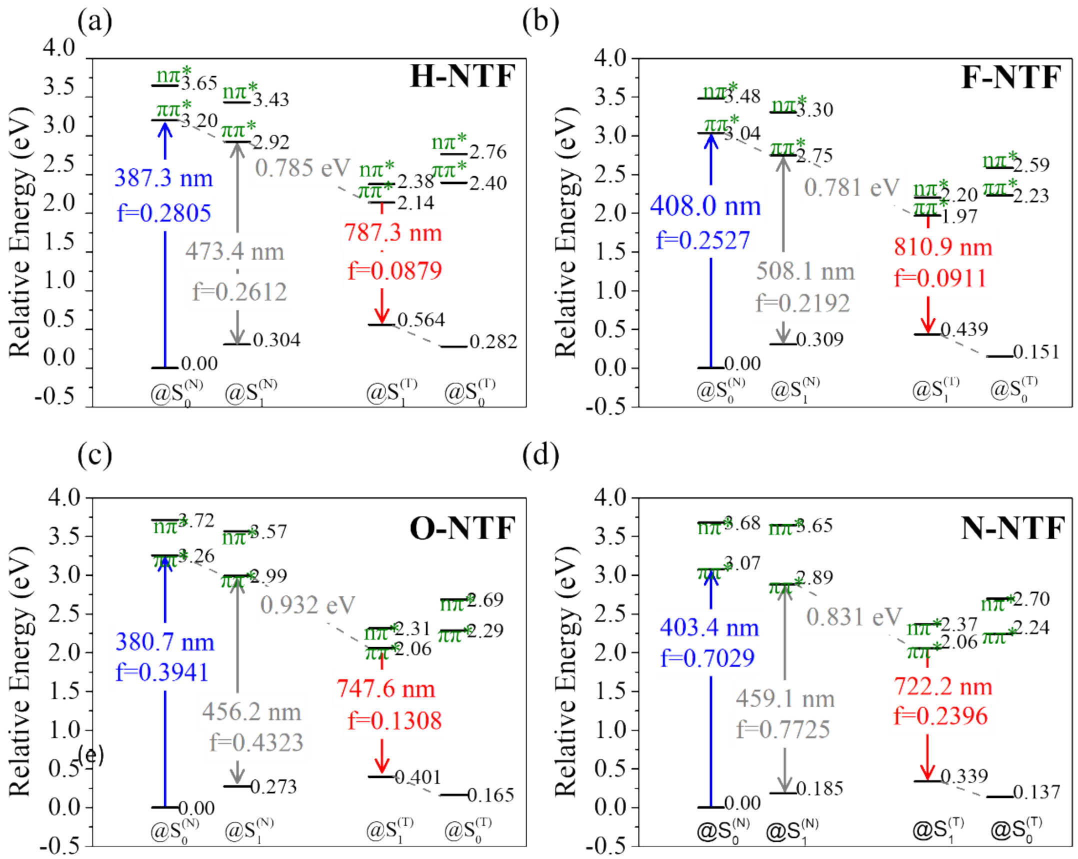

Figure S16. The calculated energy of the lower lying states and their corresponding electronic character for (a) **H-NTF** (b) **F-NTF** (c) **O-NTF** (d) **N-NTF**, where @S_0 (N), @S_1 (N) @S_0 (T) and @S_1 (T) denote the geometry optimized states at S_0 (N), S_1 (N) S_0 (T) and S_1 (T), respectively.

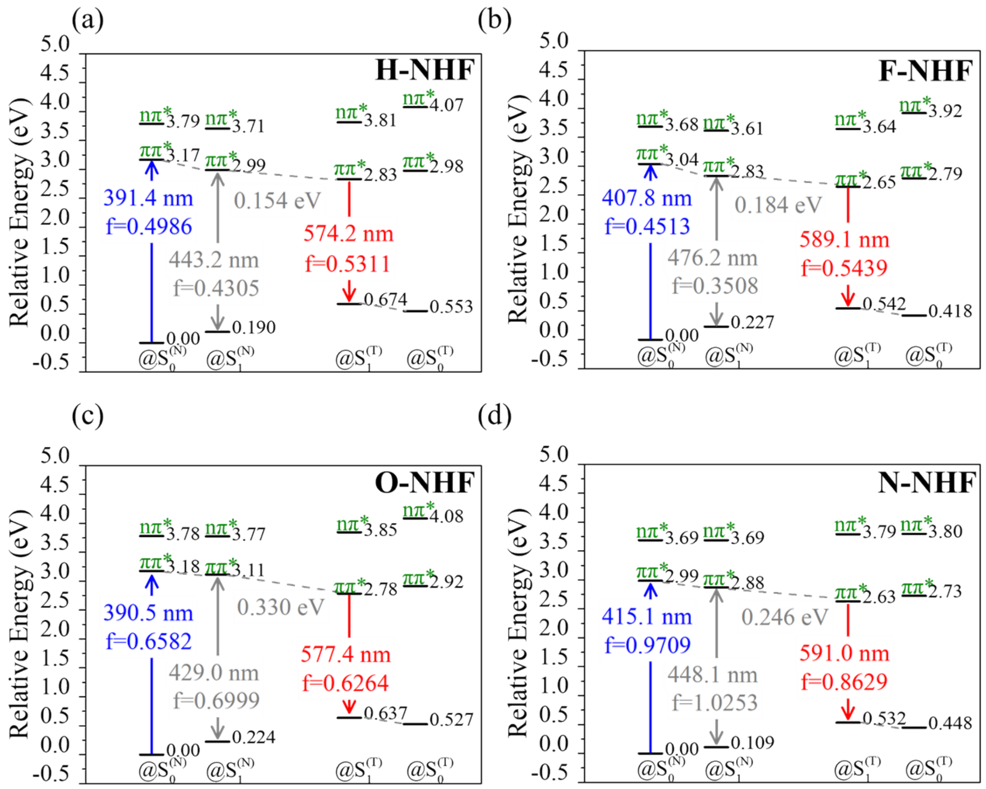

Figure S17. The calculated energy of the lower lying states and their corresponding electronic character for (a) **H-NHF** (b) **F-NHF** (c) **O-NHF** (d) **N-NHF**, where @S_0 (N), @S_1 (N) @S_0 (T) and @S_1 (T) denote the geometry optimized states at S_0 (N), S_1 (N) S_0 (T) and S_1 (T), respectively.

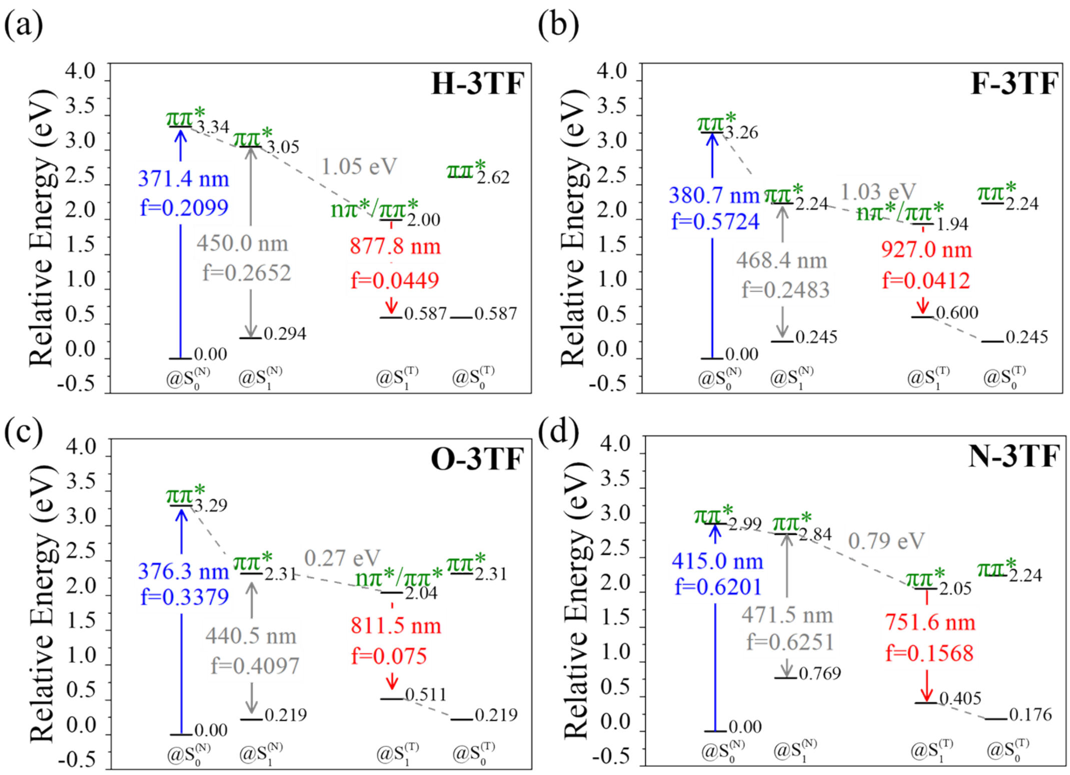

Figure S18. The calculated energy of the lower lying states and their corresponding electronic character for (a) **H-3TF** (b) **F-3TF** (c) **O-3TF** (d) **N-3TF**, where @S_0 (N), @S_1 (N) @S_0 (T) and @S_1 (T) denote the geometry optimized states at S_0 (N), S_1 (N) S_0 (T) and S_1 (T), respectively.

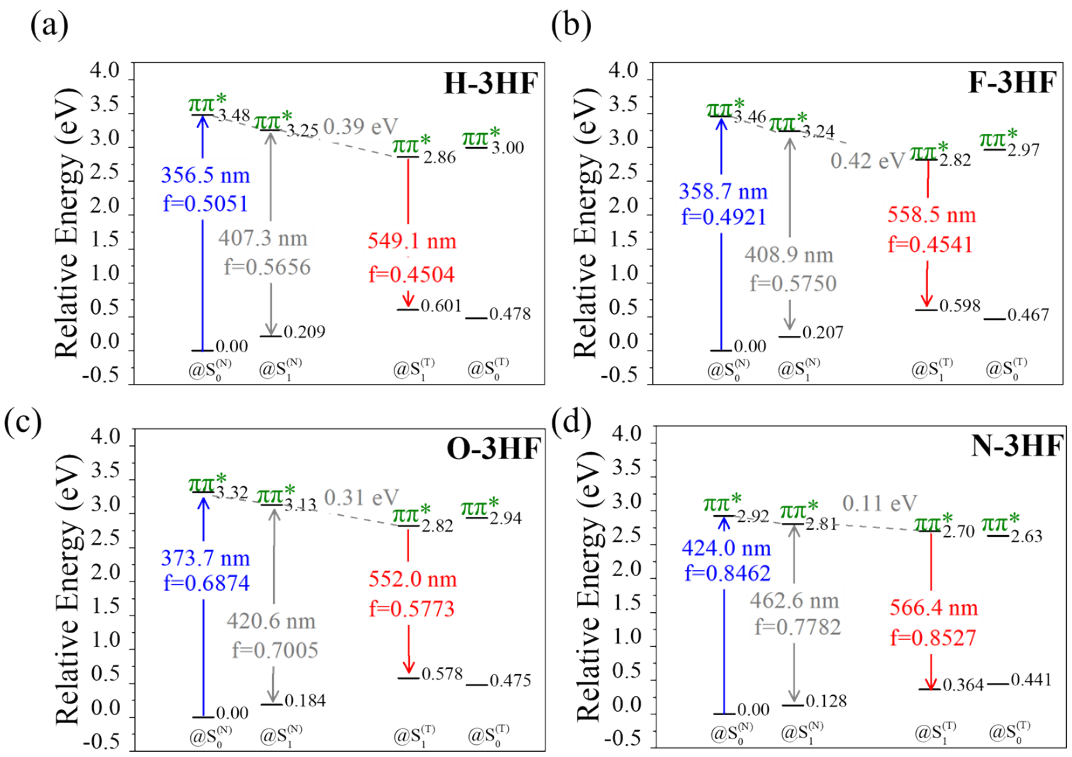

Figure S19. The calculated energy of the lower lying states and their corresponding electronic character for (a) **H-3HF** (b) **F-3HF** (c) **O-3HF** (d) **N-3HF**, where @S_0 (N), @S_1 (N) @S_0 (T) and @S_1 (T) denote the geometry optimized states at S_0 (N), S_1 (N) S_0 (T) and S_1 (T), respectively.

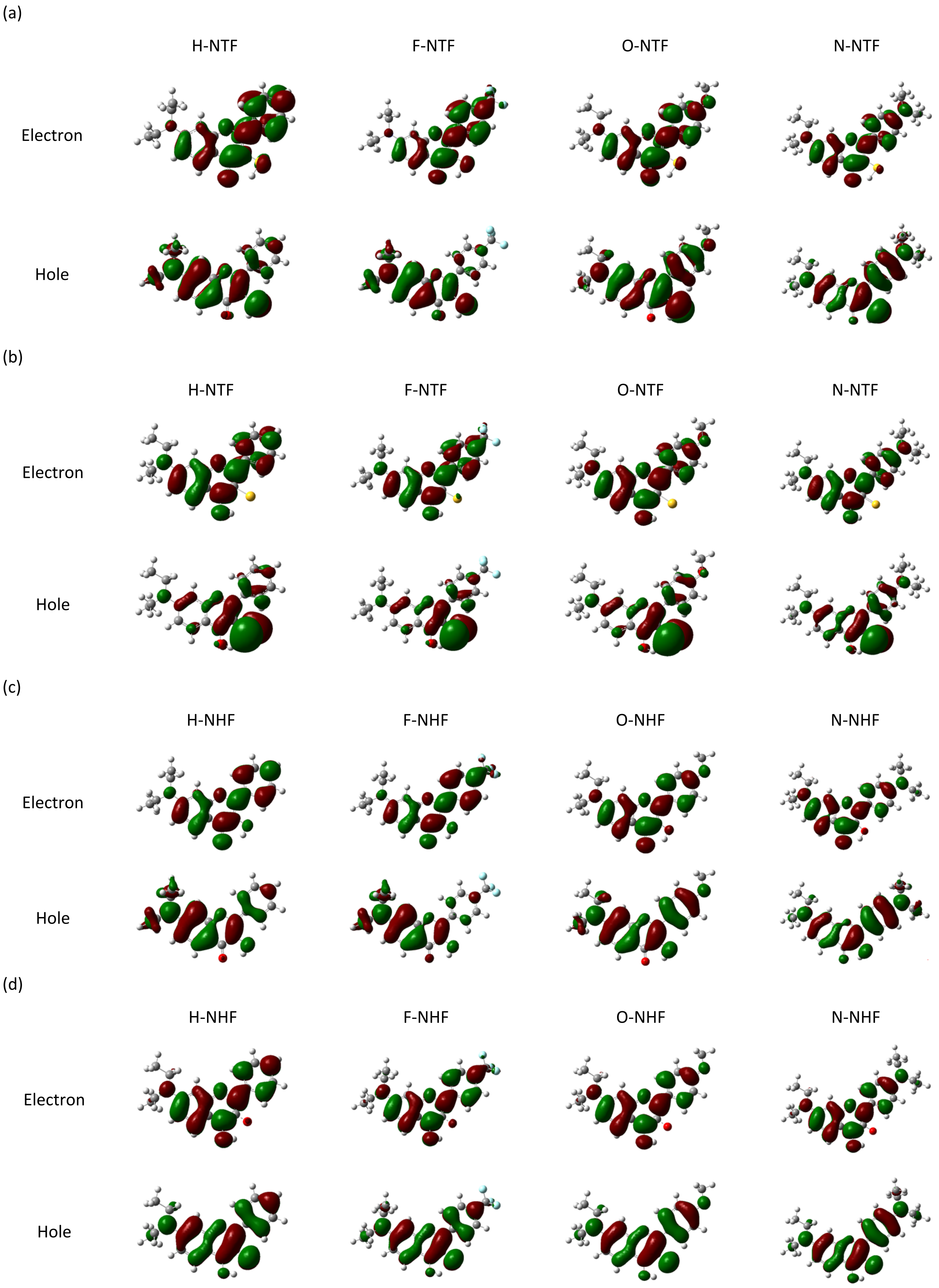

Figure S20. Frontier molecular orbitals corresponding to major optical transitions for (a) the normal form , (b) the tautomer form at the S_1-optimized structure of **NTF**s, (c) the normal form, and (d) the tautomer form at the S_1-optimized structure of **NHF**s.

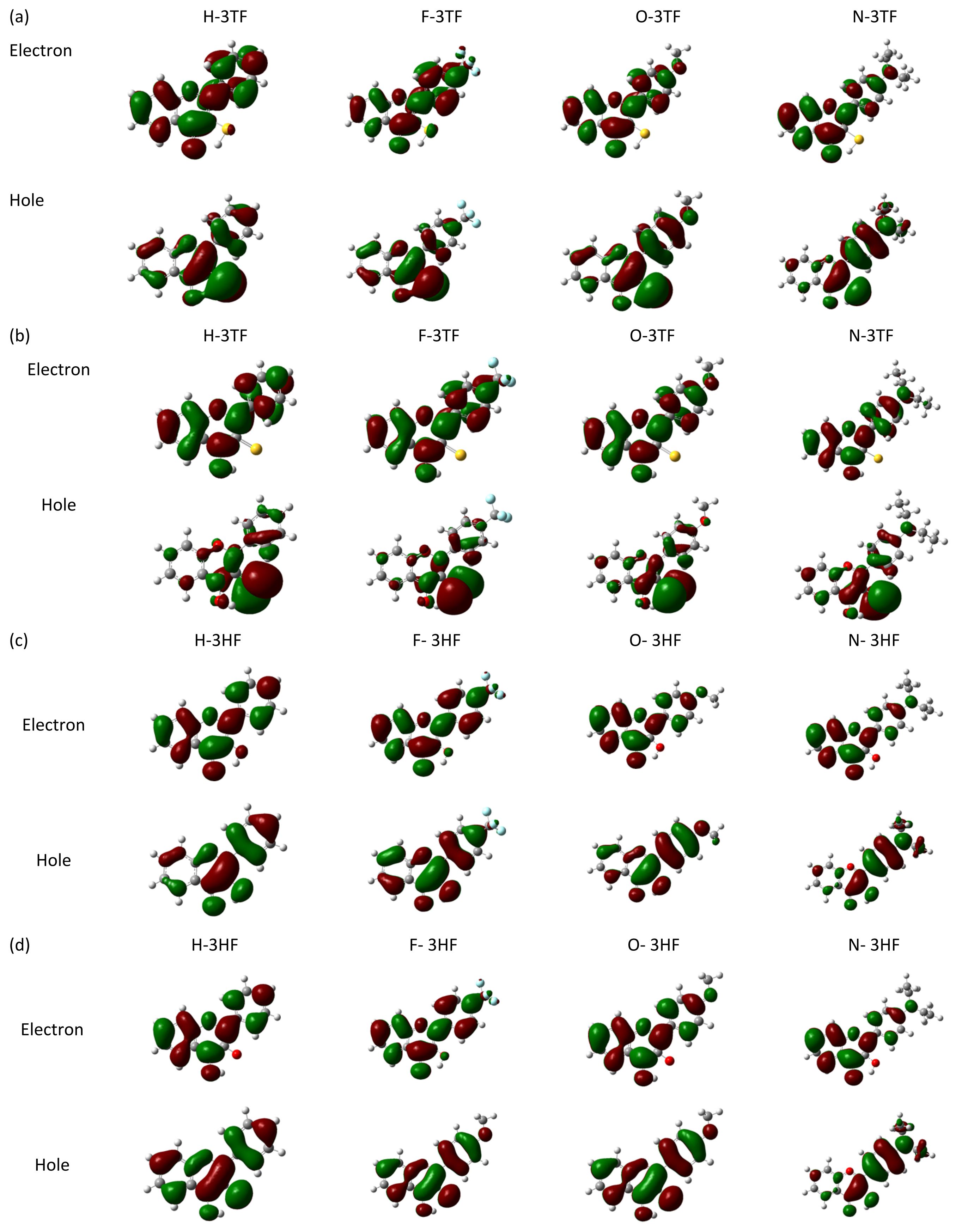

Figure S21. Natural transition orbitals of S1 for (a) the normal form at the S1-optimized structure, (b) the tautomer form at the S_1-optimized structure of **3TF**s, (c) the normal form at the S1-optimized structure, and (d) the tautomer form at the S_1-optimized structure of **3HF**s.

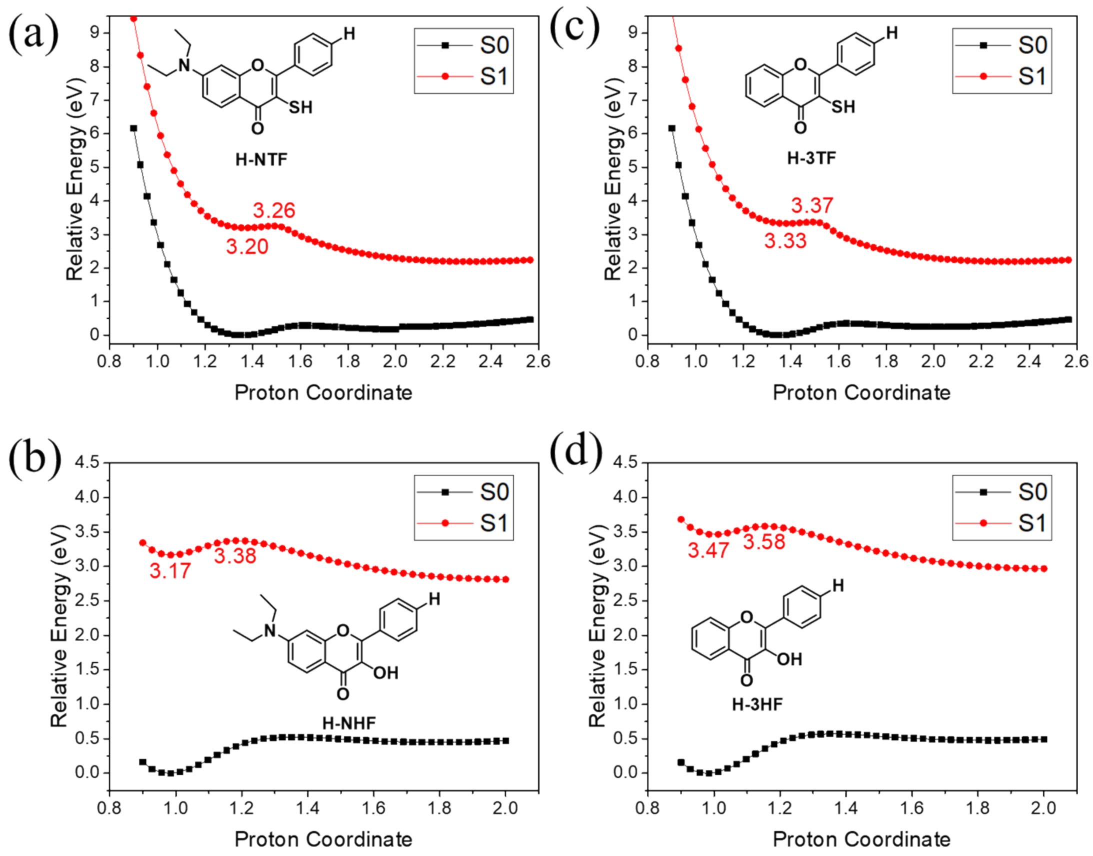

Figure S22. The potential energy surfaces of S0 and S1 along the proton coordinate in (a) **H-NTF**, (b) **H-NH**F, (c) **H-3TF**, and (d) **H-3HF**.

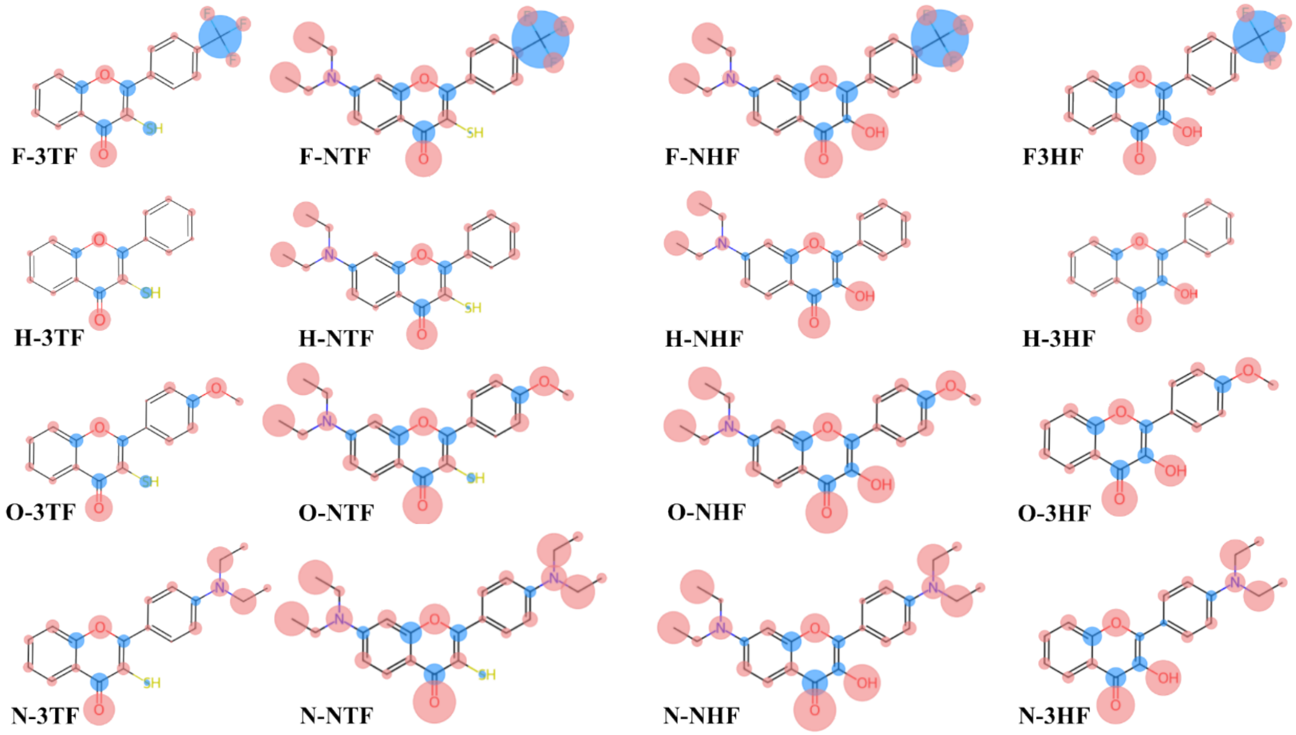

Figure S23. Visualization of Natural Population Analysis (NPA) atomic charges across ESIPT systems. Red spheres denote negative partial charges and blue spheres denote positive partial charges; sphere radius scales with charge magnitude. The figure highlights the charge redistribution patterns induced by different substituents and the contrasting behavior between sulfur- and oxygen-based donors.

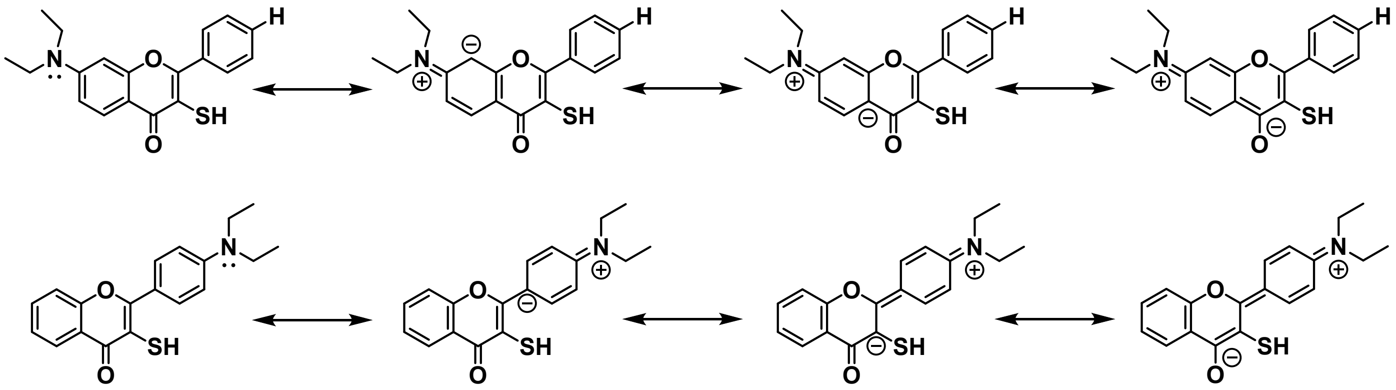

Figure S24. Schematic depiction of the resonance mechanism by which substituents modulate π-electron density across 7-N,N-diethylamino-substituted scaffold (**NTF**s and **NHF**s) and 3-scaffold (**3TF**s and **3HF**s) flavonoids, influencing ESIPT donor and acceptor sites.

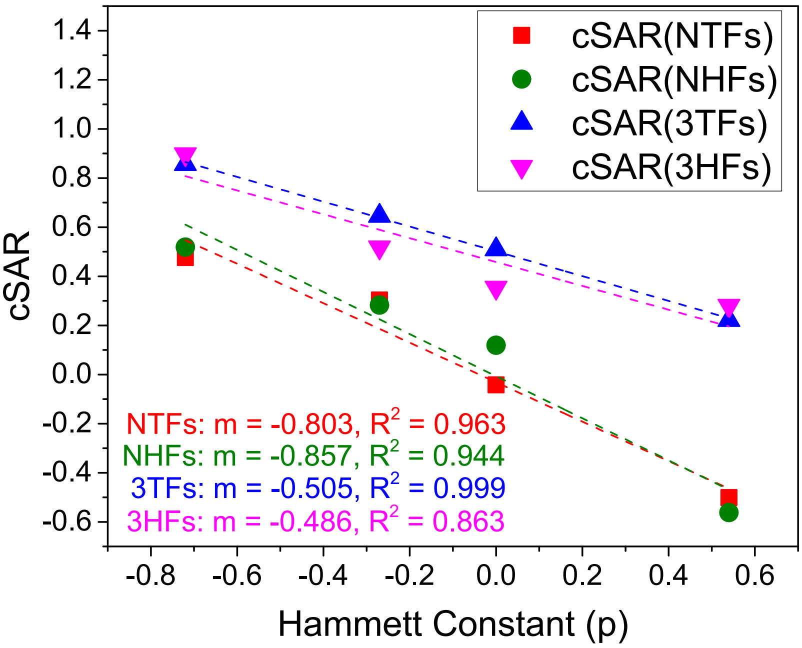

Figure S25. classical para-Hammett constants (p, here stands for σ_p) alongside the cSAR descriptors of all compounds.

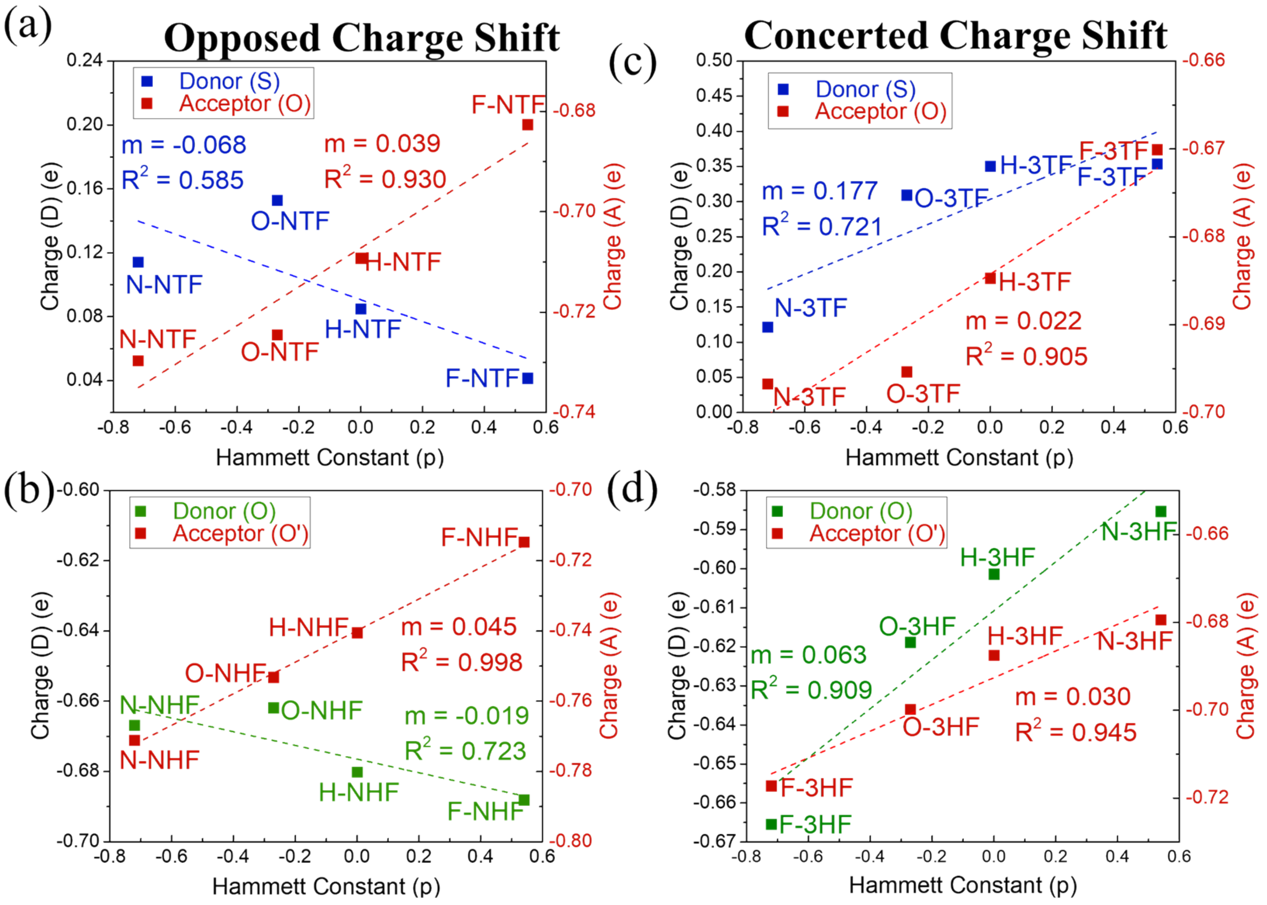

Figure S26. Correlation between the Hammett constant (p) and Natural Population Analysis (NPA) charges on the ESIPT donor and acceptor atoms across four flavonoid scaffolds: (a) **NTF**s, (b) **NHF**s, (c) **3TF**s, and (d) **3HF**s of S_1-optimized structures.. Donor atoms (S or O) are shown in blue or green; acceptor atoms (O or O’) are in red. Dashed lines represent linear fits with corresponding slopes (m) and R^2 values.

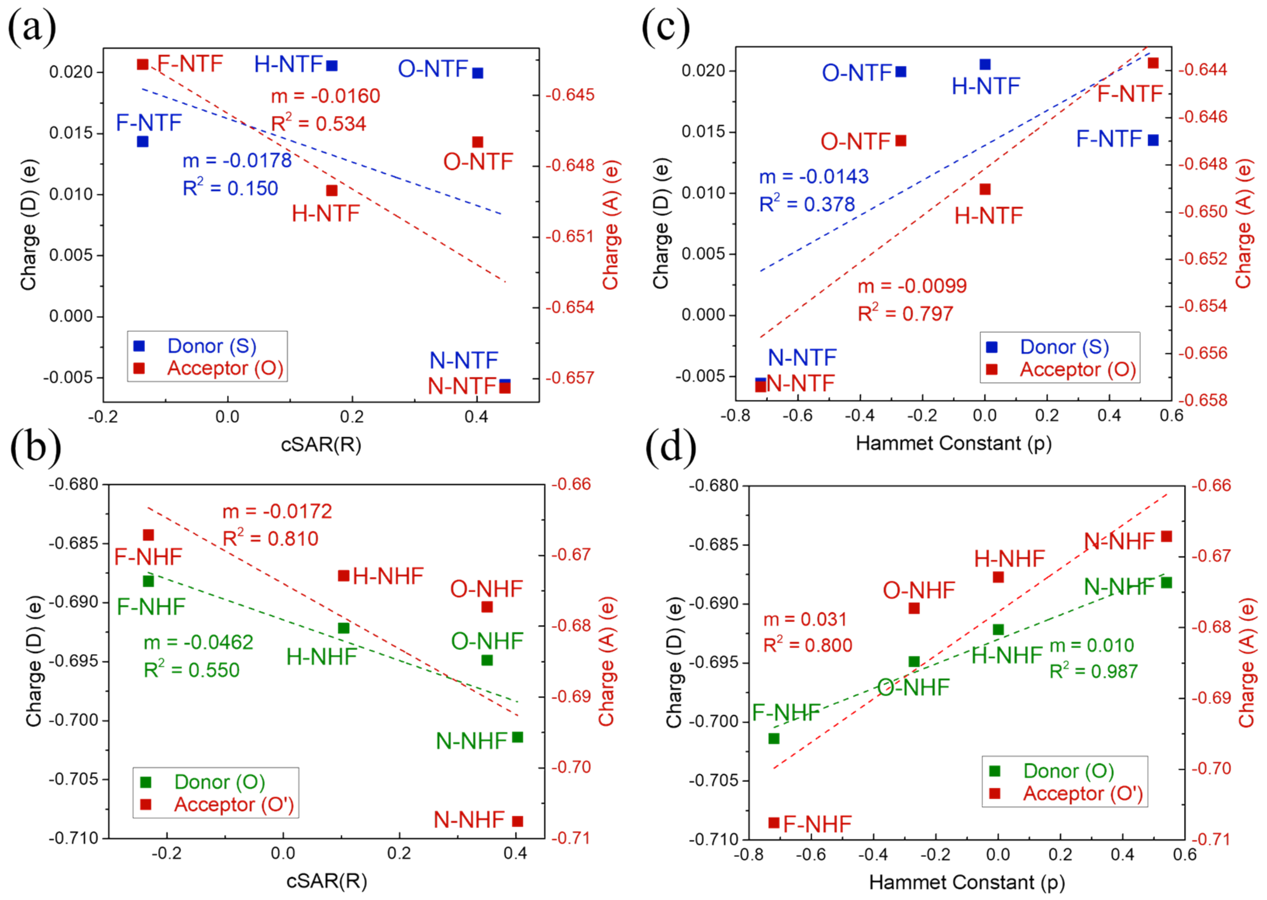

Figure S27. Correlation plots between the charge of the substituent active region (cSAR) and Natural Population Analysis (NPA) charges for (a) **NTF**s and (b) **NHF**s, and between Hammett constants (σ_p) and NPA charges for (c) **NTF**s and (d) **NHF**s of S_0-optimized structures. Donor atoms (S or O) are shown in blue or green, and acceptor atoms (O or O') in red. Dashed lines represent linear regressions with the corresponding slopes (m) and R^2 values.

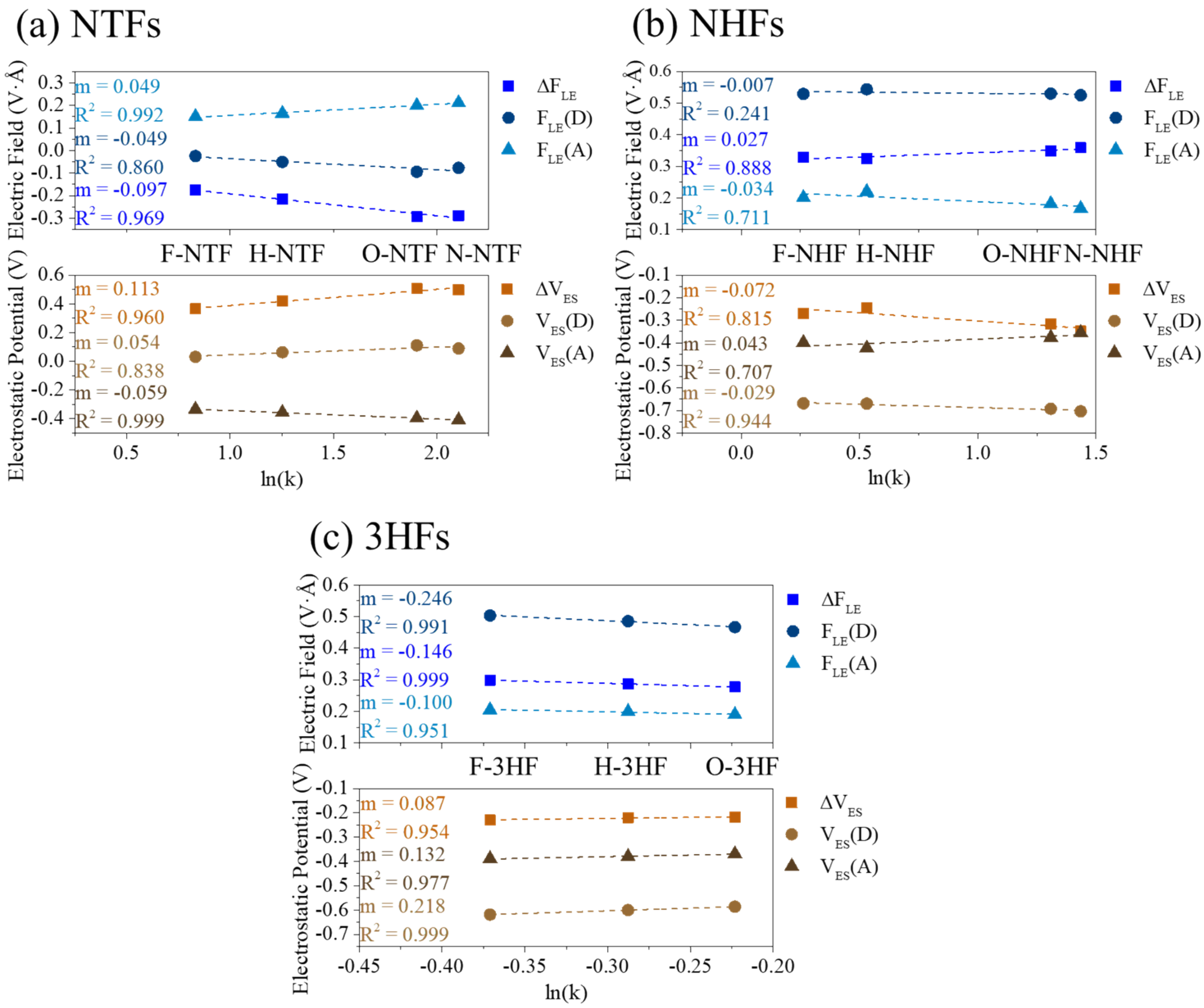

Figure S28. Correlation plots between local electrostatic descriptors and the logarithmic proton-transfer rate constant. Blue and brown schemes represent local electric field (ΔF_{LE}) and electrostatic potential (ΔV_{ES}) descriptors, respectively. Plots show linear relationships between ΔF or ΔV and ln(k) values in the S_1 state for **NTF**s, **NHF**s, and **3HF**s. Here, k denotes the proton-transfer rate constant (k_{PT}).Linear regression slopes (m) and coefficients of determination (R^2) are indicated. **3TF**s were excluded due to the absence of tautomer emission.
